# TXNIP-mediated crosstalk between oxidative stress and glucose metabolism

**DOI:** 10.1371/journal.pone.0292655

**Published:** 2024-02-08

**Authors:** Stephanie Kim, Jianning Ge, Dokyun Kim, Jae Jin Lee, Youn Jung Choi, Weiqiang Chen, James W. Bowman, Suan-Sin Foo, Lin-Chun Chang, Qiming Liang, Daiki Hara, Inpyo Choi, Myung Hee Kim, Hyungjin Eoh, Jae U. Jung

**Affiliations:** 1 Department of Molecular Microbiology and Immunology, Keck School of Medicine, University of Southern California, Los Angeles, California, United States of America; 2 Department of Cancer Biology, Infection Biology Program, and Global Center for Pathogen and Human Health Research, Lerner Research Institute, Cleveland Clinic, Cleveland, Ohio, United States of America; 3 Immunotherapy Convergence Research Center, Korea Research Institute of Bioscience and Biotechnology, Daejeon, Republic of Korea; 4 Infection and Immunity Research Laboratory, Metabolic Regulation Research Center, Korea Research Institute of Bioscience and Biotechnology, Daejeon, Republic of Korea; New York University Langone Health, UNITED STATES

## Abstract

Thioredoxin-interacting protein (TXNIP) has emerged as a key player in cancer and diabetes since it targets thioredoxin (TRX)-mediated redox regulation and glucose transporter (GLUT)-mediated metabolism. TXNIP consists of two arrestin (ARR, N-ARR and C-ARR) domains at its amino-terminus and two PPxY (PY) motifs and a di-leucine (LL) motif for endocytosis at its carboxyl-terminus. Here, we report that TXNIP shuffles between TRX and GLUTs to regulate homeostasis of intracellular oxidative stress and glucose metabolism. While TXNIP functions as a gatekeeper of TRX by default, it robustly interacted with class I GLUTs through its C-ARR domain upon increase of intracellular reactive oxygen species. This interaction prompted the surface expression downregulation and lysosomal degradation of GLUTs by its carboxyl-terminal LL endocytic signaling motif to attenuate glucose uptake. Consequently, TXNIP expression significantly limited glucose uptake, leading to the suppression of glycolysis, hexosamine biosynthesis, and the pentose phosphate pathway. Our findings establish a fundamental link between ROS and glucose metabolism through TXNIP and provide a promising target for the drug development against GLUT-related metabolic disorders.

## Introduction

Glucose is a crucial source of energy for most cells. However, due to its large size and polar nature, glucose cannot cross the cell membrane through simple diffusion and requires membrane-associated carriers. In mammals, two families of glucose transporters facilitate glucose uptake into the cells: the 14-transmembrane (14-TM) sodium-glucose linked transporters (SGLTs) and the 12-TM facilitated diffusion glucose transporters (GLUTs) [1].

Class I GLUTs is the most extensively characterized class of glucose transporters consisting of GLUT1-GLUT4 and GLUT14. GLUT1 is widely distributed and responsible for basal glucose uptake in most tissues; specifically, it is highly expressed in erythrocytes and endothelial cells of tissue barriers, playing a critical role in maintaining the low basal glucose uptake required to sustain respiration in all cells [2]. Similarly, GLUT3 and GLUT14 are high-affinity isoforms that facilitate glucose transport even in low glucose concentrations. GLUT3 is primarily expressed in neurons and the placenta, while GLUT14 is predominantly expressed in testes. GLUT4 is an insulin-regulated glucose transporter and is highly expressed in adipose tissues and striated muscles such as the skeletal and cardiac muscles. Since glucose is a crucial building block of many carbohydrates and necessary for most cells, understanding the regulation of glucose transporters, particularly class I GLUTs, in glucose uptake is crucial for studying metabolic disorders and developing therapies for related diseases.

Cancer cells require a heightened glucose supply partly due to the less efficient energy production of anaerobic glycolysis, a phenomenon known as the Warburg effect. As a result, elevated levels of GLUT1 have been observed in multiple cancer types, making it a crucial prognostic indicator for tumorigenesis. Furthermore, abnormal GLUT activity is associated with metabolic disorders such as Fanconi-Bickel Syndrome and type 2 diabetes mellitus (T2DM) [3]. Dysfunctional GLUT1 can also decrease the amount of glucose available to brain cells, affecting brain development and function. This leads to GLUT1 deficiency syndrome, characterized by early-onset seizures, microcephaly, and delayed development. Given its physiological and pathophysiological significance, there have been many research efforts focused on developing therapies targeting GLUT1.

The mammalian α-arrestin family consists of five structural arrestin domain containing proteins (ARRDC1-5) and thioredoxin interacting protein (TXNIP). Although their functions are not yet fully understood, α-arrestins are now being recognized for their roles in cancer and cellular metabolism [4]. ARRDC2 is implicated in the development and poor prognosis of ovarian cancer [5], while ARRDC3 has been linked to obesity in men and recently identified in mice as a regulator of body mass, adiposity, and energy expenditure [6]. Lastly, ARRDC4 and TXNIP are involved in regulating glucose metabolism through their interactions with GLUT1 and GLUT4 [7–9].

TXNIP is a unique member of the α-arrestin family, primarily studied for its interaction with TRX [10–12]. By forming an intermolecular disulfide bond with the reduced form of TRX, TXNIP acts as a natural inhibitor of TRX, blocking its antioxidative activity. Additionally, TXNIP also acts as a tumor suppressor [13, 14]. Overexpression of TXNIP inhibits cell proliferation and promotes apoptosis. On the other hand, reduced expression or mutation of TXNIP was found in various types of human tumors including hepatocellular carcinoma, breast cancer, bladder cancer, and leukemia [15]. Additionally, TXNIP level is markedly increased in the presence of high level of glucose, indicating a critical role of TXNIP in the regulation of glucose metabolism [16, 17]. In mice, the absence of TXNIP causes fasting hypoglycemia with a significant enhancement of glucose uptake by peripheral tissues [18], and a drug that inhibits TXNIP expression renders mice resistant to diabetes [19]. The ROS-dependent and -independent functions of TXNIP make it an attractive target for drug development in cancer and diabetes treatments, and further research on its molecular mechanism is necessary for the development of a functional and specific drug.

In this study, we hypothesize that TXNIP serves as a link between glucose metabolism and oxidative stress. TXNIP strongly interacted with GLUT1 through its C-ARR domain under conditions of increased intracellular reactive oxygen species (ROS). This interaction, mediated by three critical amino acid residues (K212, R238, and K287) on C-ARR, attenuated glucose uptake by downregulating the surface expression and inducing the lysosomal degradation of class I GLUTs. Our work discovers a fundamental connection between ROS and glucose metabolism through TXNIP and provides a promising target for drug developments against GLUT-related metabolic diseases.

## Results

### TXNIP targets glycolysis and the pentose phosphate pathway

**Fig 1:**
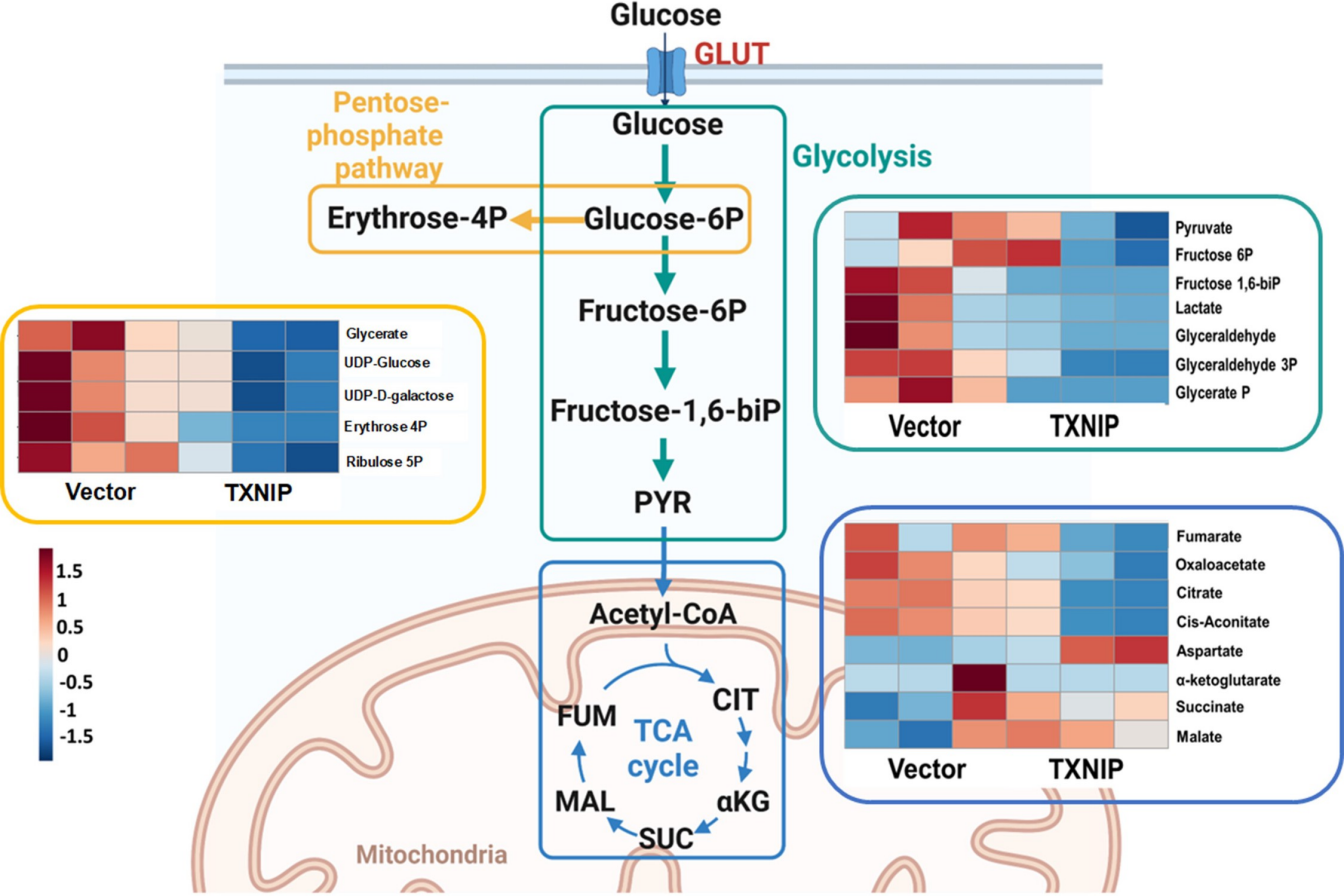
TXNIP targets glycolysis and the pentose phosphate pathway. Heat map analysis of LC-MS metabolomics. The diagram depicts glycolysis (green), pentose-phosphate pathway (yellow), and TCA cycle (blue). The GLUT is denoted in red. HEK293T cells were transduced with lentiviruses carrying vector control or TXNIP gene and were collected 48 hrs post transduction and prepared for LC-MS metabolomics. In TXNIP expressing cells, metabolites that increased were displayed in red and metabolites that decreased were displayed in blue. The heat map was created using MetaboAnalyst 5.0 (https://www.metaboanalyst.ca/MetaboAnalyst/ModuleView.xhtml) as described in Materials and methods. Glycose-6P, Glucose 6-phosphate; Erythrose 4P, Erythrose 4-phoshate; Ribulose 5P, Ribulose 5-phosphate; Fructose 6P, Fructose 6-phosphate; Fructose 1,6-biP, Fructose 1,6-bisphosphate; Glyceraldehyde 3P, Glyceraldehyde 3-phosphate; Glycerate P, Glycerate phosphate; PYR, pyruvate; CIT, citrate; αKG, α-ketoglutarate; SUC, succinate; MAL, malate; FUM, fumarate. Figure was created by BioRender.com. See also S1 Fig in S1 File.

To evaluate the role of TXNIP in glucose metabolism, HEK293T cells expressing empty vector or TXNIP were subjected to mass spectrometry mediated metabolomics (Fig 1 and S1D Fig in S1 File). The analysis was focused on the intermediates in glycolysis, the pentose phosphate pathway and the tricarboxylic acid (TCA) cycle. HEK293T-TXNIP cells showed reduced abundances of glycolysis and pentose phosphate pathway metabolites compared to those of HEK293T-Vec cells. In the glycolytic pathway, we observed significant reductions of D-Fructose 6-phosphate, D-Fructose 1,6-biphosphate, D-Glyceraldehyde 3-phosphate, and Glycerate-phosphate in HEK293T-TXNIP cells compared to HEK293T-Vec cells (Fig 1 and S1A Fig in S1 File). Consistent with glycolytic pathway intermediates, D-Erythrose 4-phosphate and D-Ribulose 5-phosphate, intermediates of the pentose phosphate pathway that utilize glucose 6-phosphate as a substrate, were also remarkably reduced in TXNIP expressing cells (Fig 1 and S1B Fig in S1 File). However, most metabolites of the TCA cycle such as α-ketoglutarate, succinate, malate, and fumarate were comparable between HEK293T-TXNIP cells and HEK293T-Vec cells (Fig 1 and S1C Fig in S1 File). These results suggest that TXNIP specifically affects metabolic pathways that directly utilize glucose such as glycolysis and the pentose phosphate pathway but not pathways further downstream such as the TCA cycle.

### TXNIP binds to and negatively regulates class I GLUT

**Fig 2:**
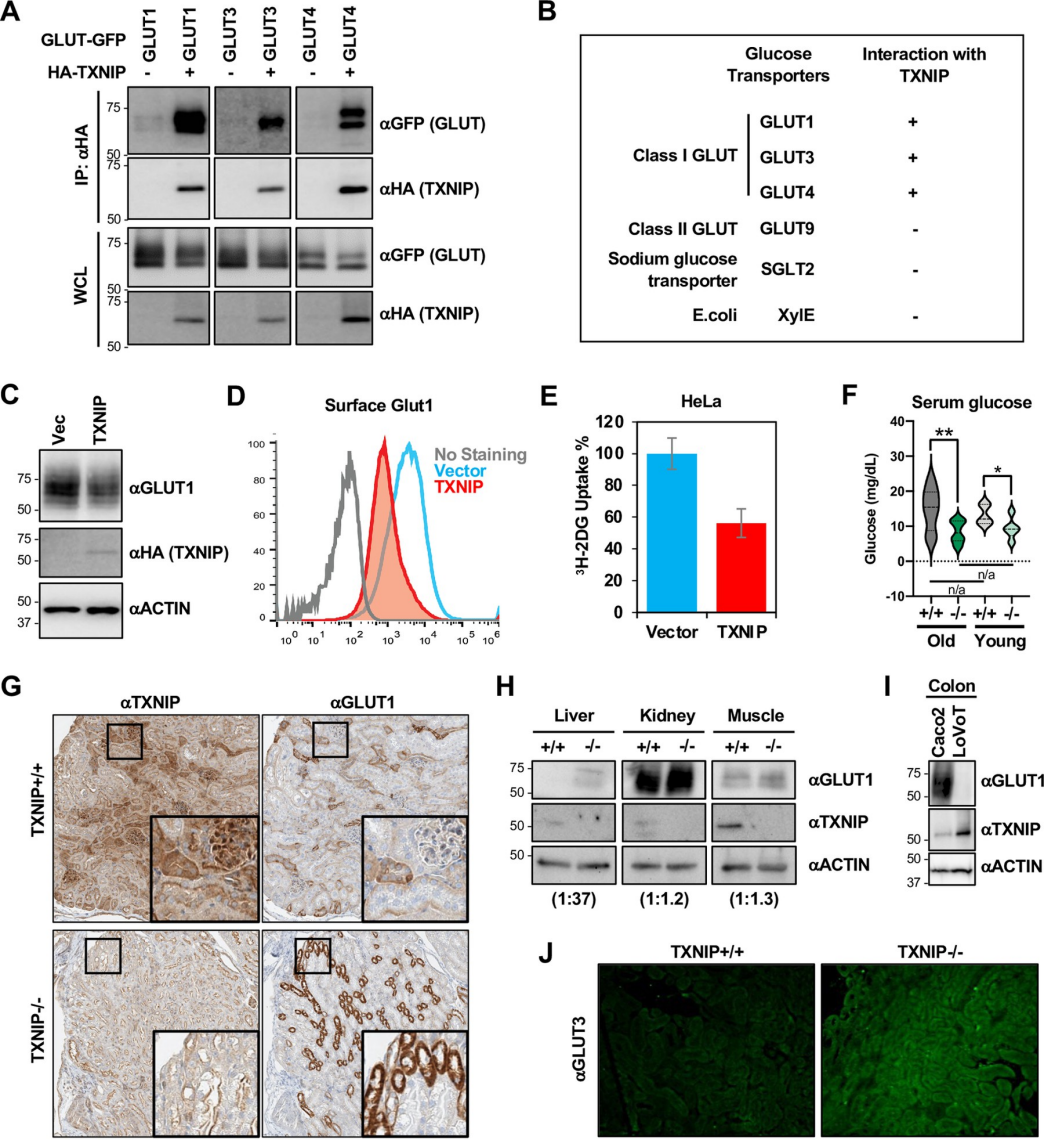
TXNIP binds to and negatively regulates class I GLUTs. (A) HEK293T cells were transfected with HA-TXNIP together with GLUT1-GFP, GLUT3-GFP or GLUT4-GFP, and HA-IP was carried out for TXNIP-GLUT interaction. (B) Summary of the interaction between TXNIP and glucose transporters of different families and different organisms. (C) HEK293T cells were transfected with vector control or TXNIP, and cell lysates were subjected to Western blot for HA, endogenous GLUT1, and ACTIN. (D) HeLa cells stably expressing HA-GLUT1 were transduced with lentiviruses carrying vector control or TXNIP gene, and surface HA-GLUT1 was examined by FACS analysis. (E) HeLa cells were transduced with lentiviruses carrying vector control or TXNIP gene, and the relative glucose uptake was examined by ^3^H-2DG tracing. Data are represented as mean ± SEM. (F) Serum glucose levels in old (11–12 months) versus young (3 months) TXNIP+/+ and TXNIP-/- mice. Statistical significance across two groups were tested by Student’s t-test; (**) p-value = 0.0100, (*) p-value = 0.0332. (G) IHC analysis of kidney slides from TXNIP+/+ and TXNIP-/- mice for GLUT1 and TXNIP. (H) Western blot analysis of GLUT1, TXNIP, and ACTIN levels in the liver, kidney, and muscle tissues from TXNIP+/+ and TXNIP-/- mice. (I) Western blot analysis of GLUT1, TXNIP, and ACTIN levels in colon cell lines, Caco2 and LoVoT. (J) Immunofluorescent staining of kidney slides from TXNIP+/+ and TXNIP-/- mice for GLUT3.

TXNIP interaction with GLUT1 was confirmed by co-immunoprecipitation (Fig 2A) and the single-molecule pull-down (SiMPull) assay (S2A Fig in S1 File). The SiMPull technique combines the principles of a conventional pull-down assay with single-molecule fluorescence microscopy and enables direct visualization of individual protein-protein interactions [20]. This method involves immobilizing protein complexes from lysed cells onto a coverslip, which is subsequently analyzed using a total internal reflection fluorescence microscope. Moreover, TXNIP demonstrated interactions with GLUT3 and GLUT4 (Fig 2A), but not with class II GLUTs (GLUT9), the mammalian 14-TM sodium glucose transporter (SGLT2), or a 12-TM bacterial glucose transporter (XyIE), indicating specific interaction of TXNIP with class I GLUTs (Fig 2B and S2B Fig in S1 File). Please refer to Table 1 for detailed material and reagents. It should be noted that the blots were exposed to different lengths of time to clearly demonstrate TXNIP-GLUT interactions. Notably, GLUT3 exhibited relatively lower enrichment in TXNIP immunoprecipitants compared to GLUT1 and GLUT4, which might be due to the difference in binding affinities to TXNIP, as GLUT-1, -3, and -4 levels were equivalent in the whole cell lysates. Furthermore, HeLa cells expressing TXNIP exhibited decreased levels of endogenous GLUT1 expression in comparison to cells expressing the vector (Fig 2C). To explore the consequence of glucose metabolism mediated by TXNIP, we co-expressed recombinant GLUT1 with an HA epitope inserted on the 1^st^ extracellular loop and TXNIP into HeLa cells. We observed a decrease in the surface expression of recombinant GLUT1 when TXNIP was co-expressed in comparison to cells co-expressing the vector (Fig 2D). This decrease in surface GLUT1 directly affected the extent of glucose uptake, as TXNIP co-expressing cells took up less glucose than vector co-expressing cells (Fig 2E). Here, 2-Deoxy-D-glucose (2-DG), a glucose analog that cannot undergo glycolysis, was used for assessment.

**Table 1:** Material and reagents.

REAGENT or RESOURCE	VENDOR	CATALOG NO.
**Antibodies**		
Mouse monoclonal HA	Biolegend	901503
Mouse monoclonal FLAG	Sigma	F1804
Mouse monoclonal ACTIN	Santa Cruz	SC-47778
Rabbit polyclonal GLUT1	Abcam	Ab15309
Rabbit polyclonal GLUT3	Abcam	Ab41525
Rabbit polyclonal TXNIP	Cell Signaling	#14715
Rabbit polyclonal TRX	Abcam	Ab86255
Rabbit monoclonal EGFR	Cell Signaling	#4267
Alexa Fluor 488	ThermoFisher	A32723
**Chemicals**		
2-DG	Sigma	D6134
^3^H-2-DG	Perkin Elmer	NET328250UC
Scintillation Cocktails	Perkin Elmer	6013681
MG-132	Sigma	474787
Baflomycin A	Invivogen	tlrl-baf1
H_2_O_2_	Sigma	216763
NAC	Sigma	A7250
Leptomycin B	Cell Signaling	#9676
Lipofectamine 2000	ThermoFisher	12566014
Fugene HD	Promega	E2311
**Recombinant DNA**		
GLUT1	Addgene	#18729
GLUT3	Addgene	#81787
GLUT4	Addgene	#18087
GLUT9	Addgene	#18730
TXNIP	Addgene	#18758
TRX	Addgene	#21615
SGLT2	GE Dharmacon	MHS6278-211690542
ARRDC4	IDT	

To evaluate the potential regulation of GLUT levels by TXNIP *in vivo*, various tissues from global TXNIP knockout (TXNIP-/-) mice and TXNIP wildtype (TXNIP+/+) mice were examined. These results revealed a marked increase of GLUT1 levels in the liver tissues of TXNIP-/- mice compared to TXNIP+/+ mice and a marginal increase of GLUT1 levels in the kidney and muscle tissues of TXNIP-/- mice compared to those of TXNIP+/+ mice (Fig 2H). The reverse correlation between GLUT1 and TXNIP expression were also observed in colon cell lines, Caco2 and LoVoT, as Caco2 cells displayed low levels of TXNIP with high levels of GLUT1, while LoVoT cells displayed high levels of TXNIP with low levels of GLUT1 (Fig 2I). Immunohistochemistry of TXNIP-/- mice and TXNIP+/+ mice kidney sections revealed apparent increases of GLUT1 in the TXNIP -/- mice compared to TXNIP+/+ mice (Fig 2G). Additionally, GLUT3 levels in the kidney tissues were higher in TXNIP-/- mice compared to TXNIP+/+ mice (Fig 2J). We also compared the overall serum glucose levels in old (11–12 months) versus young (3 months) TXNIP+/+ and TXNIP-/- mice. TXNIP-/- mice exhibited significant reduction of serum glucose levels compared to TXNIP+/+ mice regardless of age, though the decrease of glucose levels was more pronounced in old TXNIP-/- mice compared to old TXNIP+/+ mice (Fig 2F). However, there were no significant differences in serum glucose levels between old versus young TXNIP-/- mice, suggesting no age-dependent effects of TXNIP deficiency on serum glucose levels. Together, these results suggest that TXNIP interacts with class I GLUTs and this interaction downregulates GLUTs expression and glucose uptake.

### TXNIP interacts with class I GLUTs on the membrane through its central arrestin domain

**Fig 3:**
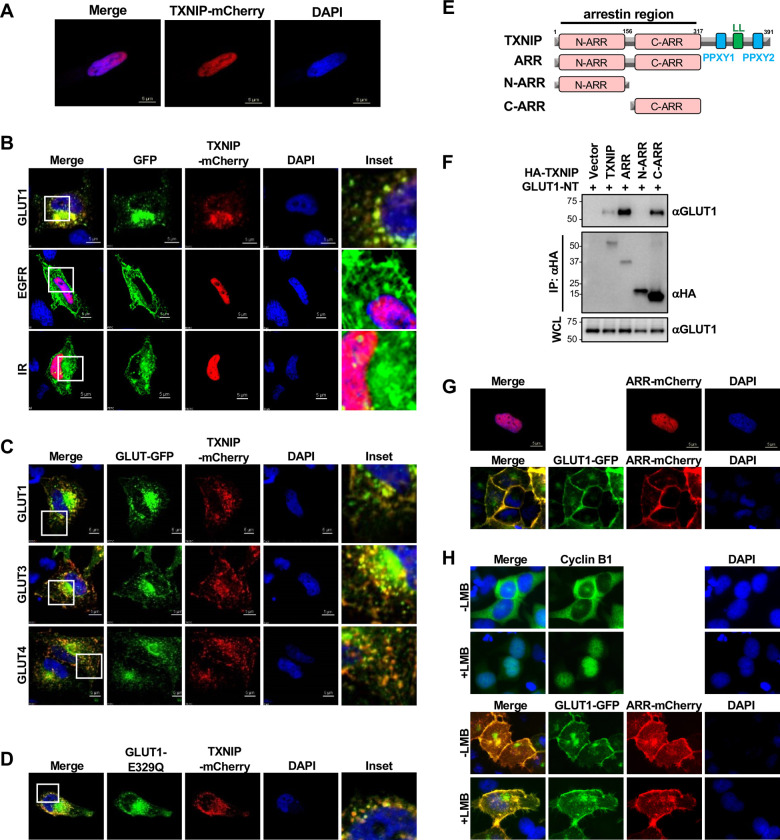
TXNIP interacts with class I GLUTs on the membrane through its central arrestin domain. (A) Confocal image of HeLa cells transfected with TXNIP-mCherry. (B) Confocal image of HeLa cells expressing TXNIP-mCherry together with GLUT1, epidermal growth factor receptor (EGFR), or insulin receptor (IR). (C) Confocal image of HeLa cells expressing TXNIP-mCherry together with GFP-fusion GLUT1, GLUT3, or GLUT4 transporter. (D) Confocal image of HeLa cells expressing TXNIP-mCherry together with GLUT1-E329Q mutation. (E) Schematics of truncated mutants of TXNIP. (F) The interaction of GLUT1 and TXNIP truncation mutants were examined through HA-IP in HEK293T cells. GLUT1-NT (GLUT1-N45T), a GLUT1 mutant which is deficient in glycosylation was used. (G) Confocal image of HeLa cells expressing TXNIP-ARR-mCherry with or without GLUT1-GFP. (H) HEK293T cells stably expressing TXNIP-ARR-mCherry and GLUT1-GFP were treated with leptomycin B (LMB) for 12 hrs and examined by fluorescent microscopy. Cyclin B1 was stained as positive control. See also S3 Fig in S1 File.

When we overexpressed TXNIP-mCherry fusion protein in HeLa cells, it displayed an exclusively nuclear localization (Fig 3A). Additionally, endogenous TXNIP was also reported to mainly localize within the nucleus of multiple cell lines [7, 21, 22]. As 12-TM GLUT family proteins primarily localize at the plasma membrane or within intracellular vesicles, we examined the effects of TXNIP and GLUT1 co-expression on their localization. Surprisingly, TXNIP-mCherry changed its localization to the plasma membrane and within intracellular puncta structures upon GLUT1 expression (Fig 3B). In addition, co-expression of GLUT3 and GLUT4 also resulted in the redirection of TXNIP to the plasma membrane and in intracellular puncta structures (Fig 3C). These changes in localization were exclusive to TXNIP and class I GLUTs co-expression since TXNIP retained its nuclear localization when co-expressed with epidermal growth factor receptor (EGFR), insulin receptor (IR), or nuclear androgen receptor (AR) (Fig 3B and S3A, S3B Fig in S1 File). In addition, class I GLUTs did not translocate to puncta structures and maintained their plasma membrane localization when co-expressed with ARRB1, a well-studied β-arrestin (S3C Fig in S1 File). To see if glucose transport played a role in this translocation, we co-expressed TXNIP-mCherry with GLUT1-E329Q, a glucose transport defective mutant [23]. Strikingly, TXNIP and GLUT1 still co-localized to the plasma membrane and in intracellular puncta structures, indicating that the glucose transport activity of GLUT1 was not required for the translocation of TXNIP (Fig 3D).

In order to test whether GLUT1 interaction was required for TXNIP translocation, we first defined the GLUT1-binding region of TXNIP by deletion mutagenesis (Fig 3E). Here, we used a mutant GLUT1, GLUT1-N45T (GLUT1-NT), which has a deficient glycosylation motif [23] and presents as a single sharp band on the blot. TXNIP contains an amino-terminal arrestin (ARR) region carrying two ARR domains (N-ARR and C-ARR) and a carboxyl-terminal region carrying two PPxY (PY) motifs and a di-leucine (LL) motif for endocytosis. Co-immunoprecipitation revealed that the ARR region of TXNIP was sufficient for strong GLUT1 interaction and that the C-ARR domain effectively bound to GLUT1, whereas the N-ARR did not (Fig 3E and 3F). Similar to TXNIP-WT, the ARR region-mCherry fusion protein translocated to the plasma membrane upon GLUT1 expression, while it primarily resided in the nucleus without GLUT1 expression (Fig 3G). Since previous reports found that the importin system mediates the nuclear import of TXNIP [22], we next assessed if TXNIP interacts with GLUTs prior to its nuclear import. When a potent nuclear export inhibitor leptomycin B (LMB) was used to block the nuclear export process, TXNIP was still able to co-localize with GLUT1 on the cell surface, while the nuclear export of cyclin B1 was fully blocked under the same conditions (Fig 3H). Collectively, these data indicate that TXNIP binds to class I GLUTs through its C-terminal ARR and directly traffics to the plasma membrane upon GLUTs expression.

### TXNIP interaction mediates the endocytic trafficking of GLUT1

**Fig 4:**
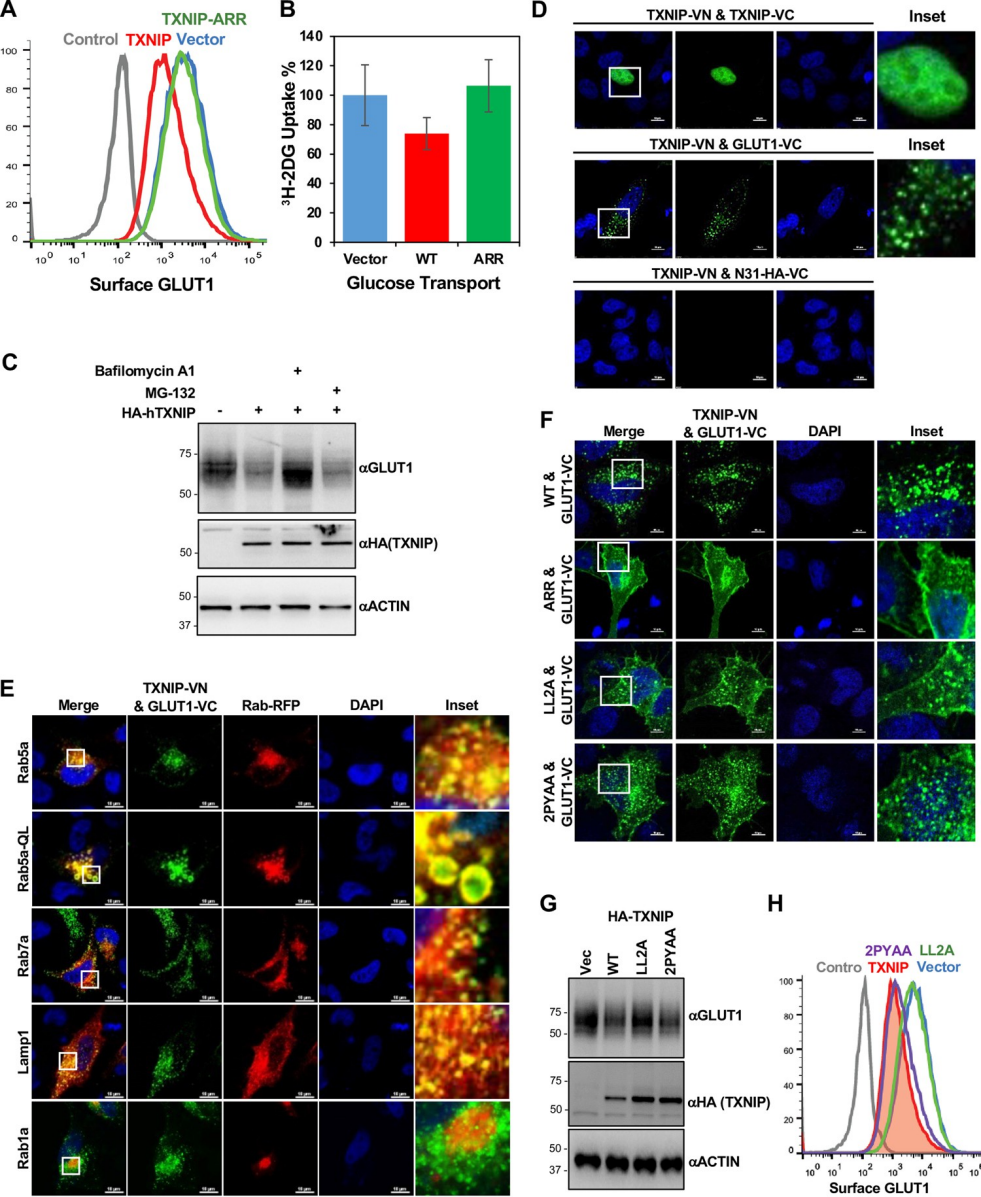
TXNIP interaction mediates the lysosomal degradation of GLUT1. (A) HeLa cells stably expressing HA-GLUT1 were infected with lentiviruses carrying vector control, TXNIP gene, or TXNIP-ARR gene, and surface HA-GLUT1 was examined by FACS analysis. (B) HeLa cells were transduced with lentiviruses carrying vector control, TXNIP gene, or TXNIP-ARR gene, and relative glucose uptake was examined by ^3^H-2DG tracing. Data are represented as mean ± SEM. (C) HeLa cells expressing vector control or TXNIP were treated with MG-132 or bafilomycin A1 for 12 hrs, and the cell lysates were subjected to GLUT1, HA, and EGFR blots. (D) Confocal image of HeLa cells expressing TXNIP-VN together with TXNIP-VC, GLUT1-VC, or N31-HA-VC. Green fluorescent signal represents VN&VC complexes. (E) Confocal image of HeLa cells expressing TXNIP-VN & GLUT1-VC with endosomal markers Rab5a, Rab5a-QL, Rab7a, or lysosomal Lamp1 as well as Golgi marker Rab1a. (F) Confocal image of HeLa cells expressing GLUT1-VC together with TXNIP-VN, TXNIP-ARR-VC, TXNIP-LL2A-VC, or TXNIP-2PYAA-VC. (G) HeLa cells expressing vector control, TXNIP, TXNIP-LL2A mutant, or TXNIP-2PYAA mutant were examined by Western blot for the expression of endogenous GLUT1, HA and ACTIN. (H) HeLa cells stably expressing HA-GLUT1 were transduced with lentiviruses carrying vector control, TXNIP gene, TXNIP-LL2A gene, or TXNIP-2PYAA gene, and surface HA-GLUT1 was examined by FACS analysis.

Interestingly, while the ARR region of TXNIP was sufficient for strong GLUT1 interaction, it alone was not able to downregulate the surface expression and glucose transport activity of GLUT1 (Fig 4A and 4B). In addition, TXNIP-mediated reduction of GLUT1 level was blocked by bafilomycin A1 lysosome inhibitor, but not by MG-132 proteasome inhibitor (Fig 4C). To specifically capture the interaction between TXNIP and GLUT1, we utilized a bimolecular fluorescence complementation (BiFC) assay (S4A Fig in S1 File). Co-expression of TXNIP-VN and TXNIP-VC showed a specific nuclear green fluorescence signal, consistent with previous reports that TXNIP forms an interprotomer-disulfide bond-mediated dimer [24, 25], whereas co-expression of TXNIP-VN and an unrelated protein N31-VC did not show any fluorescent signals (Fig 4D). When TXNIP-VN and GLUT1-VC were co-expressed, distinct intracellular vesicle-shaped green fluorescent signals were captured, with little to no co-localization on the plasma membrane. Furthermore, those intracellular vesicle-shaped green fluorescent signals of TXNIP-VN and GLUT1-VC considerably overlapped with signals of red fluorescence protein (RFP)-Rab5, -Rab7 or -LAMP1, but not with those of RFP-Rab1a (Fig 4E). Specifically, expression of the constitutively active Rab5-QL mutant generated large intracellular vesicles, with the TXNIP-VN/GLUT1-VC complexes extensively co-localized within these vesicles, primarily on their surfaces (Fig 4E). These results indicate that the TXNIP-GLUT1 complexes undergo endocytosis and travel to the lysosomal compartments.

To further delineate the intracellular localization of TXNIP-GLUT1 complexes, the BiFC system was also used on TXNIP mutants: the amino-terminal ARR only mutant (ARR-VN), the LL2A mutant carrying a replacement of di-leucine motif with alanine (LL2A-VN), and the 2PYAA mutant carrying a replacement of the two PY motifs with alanine (2PYAA-VN) (S4B Fig in S1 File). CellMask was included to allow uniform labeling of the plasma membrane (S4C Fig in S1 File). The ARR-VN/GLUT1-VC complexes were present extensively at the plasma membrane (Fig 4F and S4C Fig in S1 File). Compared to the TXNIP-VN/GLUT1-VC complexes, the LL2A-VN/GLUT1-VC complexes primarily showed plasma membrane localization and limited intracellular vesicle localization (Fig 4F and S4C Fig in S1 File). On the other hand, the 2PYAA-VN/GLUT-VC complexes showed intracellular vesicle localization similar to the TXNIP-WT-VN/GLUT1-VC complexes (Fig 4E and S4C Fig in S1 File). Furthermore, while both TXNIP-WT and 2PYAA mutant clearly downregulated the surface expression and overall levels of GLUT1, the LL2A mutant did not (Fig 4G and 4H). Collectively, these results suggest that the N-terminal ARR region of TXNIP is responsible for GLUT1 interaction and the C-terminal di-leucine motif of TXNIP plays critical roles in the endocytosis and degradation of GLUT1.

### ARRDC4 effectively targets and downregulates class I GLUTs

**Fig 5:**
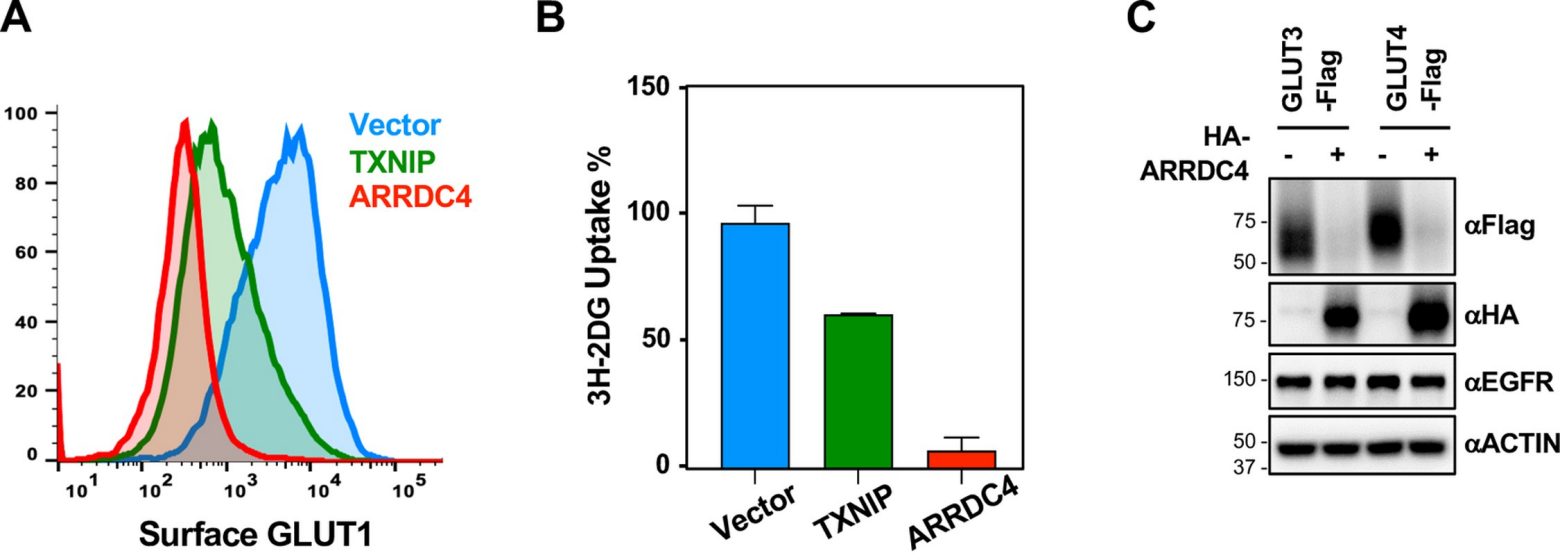
ARRDC4 effectively targets and downregulates class I GLUTs. (A) HeLa cells stably expressing HA-GLUT1 were transduced with lentiviruses carrying vector control, TXNIP, or ARRDC4 genes, and surface HA-GLUT1 was examined by FACS analysis. (B) HeLa cells were transduced with lentiviruses carrying vector control, TXNIP, or ARRDC4 genes, and the relative glucose uptake was examined by ^3^H-2DG tracing. Data are represented as mean ± SEM. (C) HEK293T cells stably expressing GLUT3-flag or GLUT4-flag were transduced with a lentivirus encoding ARRDC4, and cell lysates were collected and analyzed by Western blot for Flag, HA, EGFR, and ACTIN.

Two members of the α-arrestin superfamily, TXNIP and ARRDC4, have been previously reported to bind to and downregulate GLUT1. In fact, ARRDC4 dramatically reduced the surface expression and glucose transport activity of GLUT1 much more potently than TXNIP (Fig 5A and 5B). Moreover, ARRDC4 also effectively targeted and downregulated GLUT3 and GLUT4 (Fig 5C). In addition, ARRDC4’s activity was specific to class I GLUTs, as it had no impact on the expression of EGFR (Fig 5C). These results indicate that both TXNIP and ARRDC4 represents members of the α-arrestin protein family that targets and downregulates the class I GLUT family.

### Thioredoxin efficiently competes with GLUT1 for TXNIP interaction

**Fig 6:**
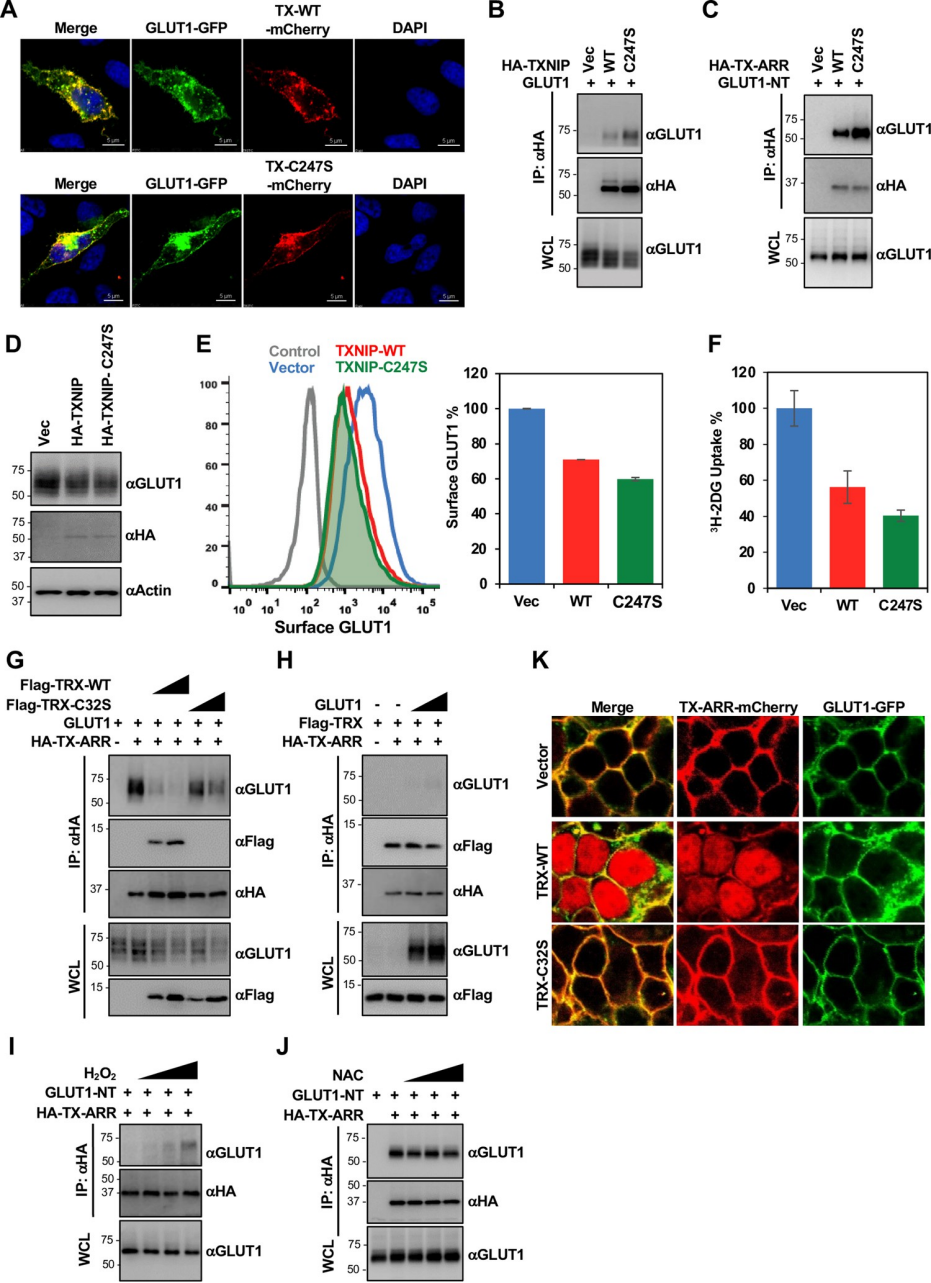
Thioredoxin efficiently competes with GLUT1 for TXNIP interaction. (A) Confocal image of HeLa cells expressing GLUT1-GFP together with TXNIP-WT-mCherry or TXNIP-C247S-mCherry. (B) HEK293T cells were transfected with GLUT1 and vector control, HA-TXNIP-WT, or HA-TXNIP-C247S. HA-IP was carried out to determine the interaction between TXNIP and GLUT1. (C) HEK293T cells were transfected with GLUT1 and vector control, HA-TXNIP-ARR-WT, or HA-TXNIP-ARR-C247S. HA-IP was carried out to determine the interaction between TXNIP-ARR and GLUT1. (D) HeLa cells were transduced with lentiviruses carrying vector control, TXNIP gene, or TXNIP-C247S gene. Cell lysates were examined for endogenous GLUT1, HA, and ACTIN. (E) (Left) HeLa cells stably expressing HA-GLUT1 were transduced with lentiviruses carrying vector control, TXNIP gene, or TXNIP-C247S gene, and surface HA-GLUT1 was examined by FACS analysis. (Right) Quantification of surface HA-GLUT1 on the left panel. Data are represented as mean ± SEM. (F) HeLa cells were transduced with lentiviruses carrying vector control, TXNIP gene, or TXNIP-C247S gene, and relative glucose uptake was examined by ^3^H-2DG tracing. Data are represented as mean ± SEM. (G) HeLa cells were transfected as indicated with different doses of TRX or TRX-C32S, and HA-IP was carried out to determine the interaction between TXNIP-ARR and GLUT1. (H) HeLa cells were transfected as indicated with increased amount of GLUT1, and HA-IP was carried out to determine the interaction between TXNIP-ARR and TRX. (I-J) HEK293T cell expressing GLUT1 and HA-TXNIP-ARR was treated with (I) H_2_O_2_ (30 μM, 300 μM, 1.5 mM) or (J) NAC (2.5 μM, 5 μM, 10 μM) for 4 hrs, and HA-IP was carried out to determine the interaction between GLUT1 and TXNIP-ARR. (K) HEK293T cells stably expressing GLUT1-GFP and TXNIP-ARR-mCherry were transfected with TRX-WT or TRX-C32S, and the colocalization of GLUT1 and TXNIP were examined by confocal microscope.

Since TXNIP forms a ROS-sensitive covalent disulfide bond with TRX, we used a TXNIP-C247S mutant that no longer forms a disulfide bond with TRX [3] to see how this affects GLUT1 interaction. Similar to TXNIP-WT, the TXNIP-C247S mutant also showed clear co-localization with GLUT1 at the plasma membranes and in intracellular vesicles (Fig 6A). In fact, the TXNIP-C247S mutant showed higher GLUT1 binding and degradation activity than TXNIP-WT (Fig 6B and 6D), resulting in a more significant reduction in surface expression and glucose transport activity of GLUT1 (Fig 6E and 6F). On the other hand, while both the wildtype arrestin domain and C247S mutation in the arrestin domain (ARR-C247S) did not reduce the intracellular amount of GLUT1, ARR-C247S showed much higher GLUT1 binding activity (Fig 6C).

Next, we examined if the strong disulfide interaction between TRX and TXNIP can affect the interaction between TXNIP and GLUT1. We used the TXNIP ARR fragment (TX-ARR) since it interacted with TRX and GLUT1 as efficiently as TXNIP-WT and did not degrade GLUT1. Increasing expression of TRX-WT, but not TRX-C32S mutant that no longer forms a disulfide interaction with TXNIP, led to reduced TX-ARR interaction with GLUT1 (Fig 6G), whereas increasing the expression of GLUT1 showed no effect on TX-ARR interaction with TRX (Fig 6H). These data indicated efficient competition of TRX with GLUT1 for TXNIP’s interaction. Because a covalent disulfide interaction between TX-ARR and TRX is sensitive to ROS [24], we checked the effects of ROS inducer (H_2_O_2_) and ROS inhibitor [N-acetyl-L-cysteine (NAC)] on TX-ARR interaction with GLUT1. Treatment with increasing concentrations of H_2_O_2_, serving as conditions that mimic increasing ROS levels, led to the marked increase of TX-ARR interaction with GLUT1, whereas NAC treatment led to the slight decrease of TX-ARR interaction with GLUT1 (Fig 6I and 6J). To test TRX’s effect on the co-localization of TXNIP-GLUT1, HeLa cells stably expressing GLUT1-GFP and TX-ARR-mCherry were transfected with vector, TRX-WT, or TRX-C32S. While GLUT1-GFP and TX-ARR-mCherry effectively co-localized at the plasma membrane, TRX-WT expression resulted in the nuclear localization of TX-ARR-mCherry (Fig 6K). By striking contrast, TRX-C32S mutant expression showed no effect on TX-ARR-mCherry as TX-ARR-mCherry remained at the plasma membrane with GLUT1-GFP (Fig 6K). These results collectively indicate that TRX efficiently outcompetes GLUT1 for TXNIP interaction, which can be reversed by an ROS inhibitor.

### The positively charged interface of TXNIP central arrestin domain is essential for GLUT1 interaction and glucose metabolism

**Fig 7:**
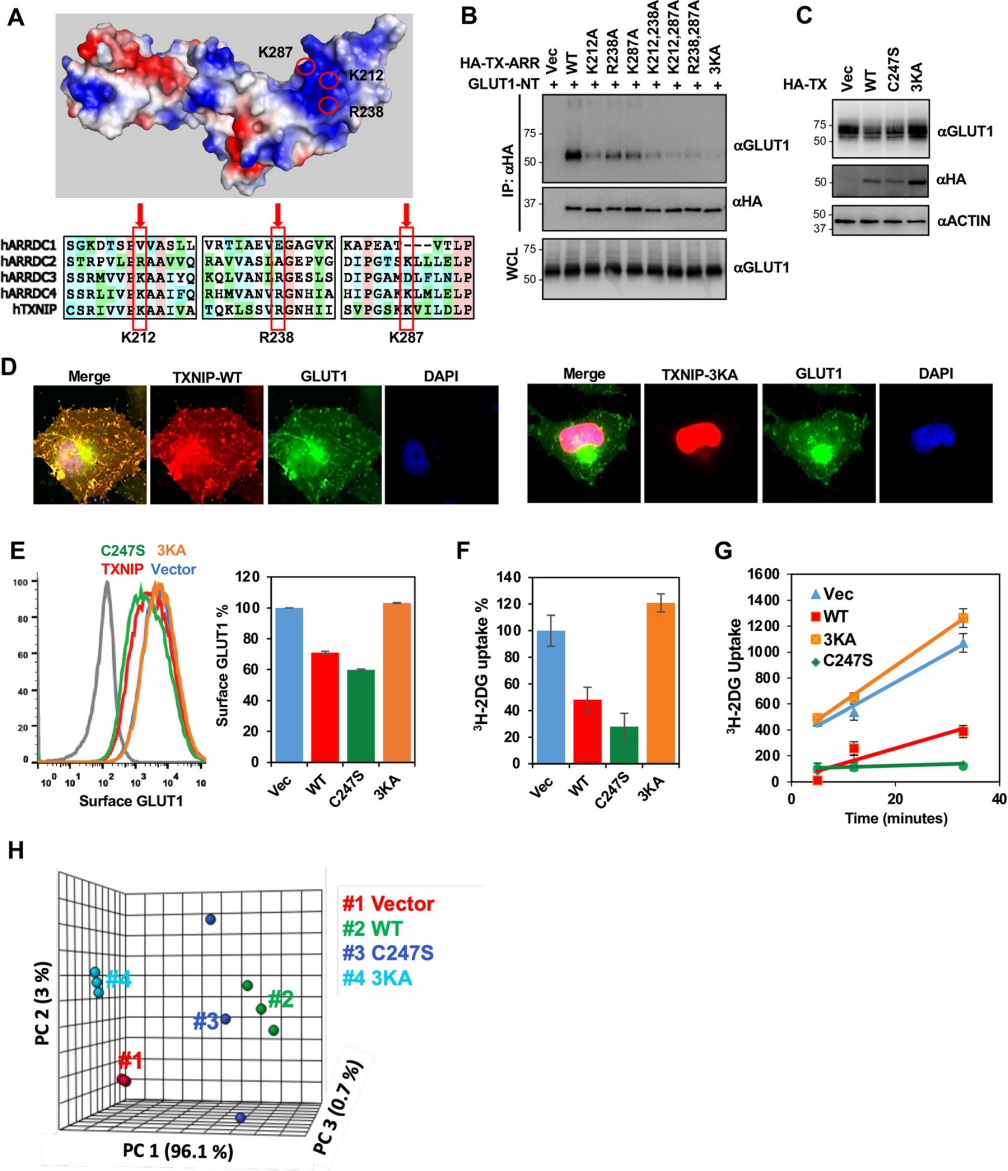
The positively charged interface of TXNIP central arrestin domain is essential for GLUT1 interaction and glucose metabolism. (A) (Top) Schematic figure demonstrates the group of positively charged residues located in the central arrestin domain of TXNIP. (PDB: 4LL1) (Bottom) Sequence alignment of alpha-arrestin family members shows conserved arrangement of positively charged residues in the central arrestin domain. (B) HeLa cells were transfected as indicated, and HA-IP was carried out to determine the interaction between GLUT1 and TXNIP-ARR mutants. (C) HeLa cells were transduced with lentiviruses carrying vector control, TXNIP, TXNIP-C247S, or TXNIP-3KA genes, and cell lysates were examined for endogenous GLUT1, HA, and ACTIN. (D) Confocal image of HeLa cells expressing GLUT1-GFP and TXNIP-WT-mCherry or TXNIP-3KA-mCherry. (E) (Left) HeLa cells stably expressing HA-GLUT1 were transduced with lentiviruses carrying vector control, TXNIP-WT, TXNIP-C247S, or TXNIP-3KA genes, and surface HA-GLUT1 was examined by FACS analysis. (Right) Quantification of surface HA-GLUT1 on the left panel. Data are represented as mean ± SEM. (F) HeLa cells were transduced with lentiviruses carrying vector control, TXNIP, TXNIP-C247S, or TXNIP-3KA genes, and the relative glucose uptake was examined by ^3^H-2DG tracing. Data are represented as mean ± SEM. (G) Kinects of ^3^H-2DG uptake in HeLa cells expressing vector control, HA-TXNIP-WT, HA-TXNIP-C247S, or HA-TXNIP-3KA. (^3^H-2DG uptake were examined at 5 min, 12 min and 33 min after adding ^3^H-2DG). Data are represented as mean ± SEM. (H) Principal Component Analysis (PCA) of LC-MS metabolomics in HEK293T cells expressing vector control, TXNIP-WT, TXNIP-C247S, or TXNIP-3KA.

The crystal structure of TXNIP indicates that its central arrestin domain is enriched with basic amino acids, including K212, R238 and K287 residues that are highly conserved among ARRDC2, ARRDC3 and ARRDC4, members of the α-arrestin family (Fig 7A). Indeed, alanine replacement mutation at K212, R238 or K287 of TXINP detectably reduced GLUT1 interaction, whereas triple mutation (3KA) of TXNIP carrying the replacements of K212, R238 and K287 with alanine completely abolished GLUT1, GLUT3 and GLUT4 interaction (Fig 7B, S5A, S5B Fig in S1 File). Consistently, the TXNIP-3KA mutant neither co-localized with GLUT1 nor induced the reduction of GLUT1 levels and surface expression (Fig 7C–7E). Furthermore, the TXNIP-3KA mutant did not affect glucose uptake and kinetics (Fig 7F and 7G). To identify the metabolic consequences of impaired GLUT1 interaction with TXNIP-3KA, we conducted metabolomics. Principal Component Analysis (PCA) showed that expression of TXNIP-WT or TXNIP-C247S led to similar effects on cellular metabolic homeostasis (Fig 7H and S5A Fig in S1 File). Glycolysis (S5B Fig in S1 File), hexosamine biosynthesis pathway (S5C Fig in S1 File), and pentose phosphate pathway (S5D Fig in S1 File) were dramatically suppressed in both TXNIP-WT and TXNIP-C247S. However, expression of TXNIP-3KA mutant lost the foregoing effect on overall metabolisms (S5A-S5D Fig in S1 File). Finally, no obvious alterations in the TCA cycle were detected by expression of TXNIP-WT, TXNIP-C247S or TXNIP-3KA (S5E Fig in S1 File). These results collectively show that the positively charged interface of TXNIP central arrestin domain is essential for GLUT1 interaction and thereafter the alteration of glucose metabolism. This also suggests that TXNIP shuffles between TRX and GLUT1 in a genetically separable manner.

## Discussion

The α-arrestin proteins are emerging as a family of proteins that regulate metabolism and the development of obesity. TXNIP and ARRDC4 have been implicated in the regulation of glucose metabolism and ARRDC3 is linked to male-specific association with high body mass index (BMI) in humans [6, 7]. Distinct from members of the α-arrestin family, TXNIP also binds to TRX to suppress its antioxidative function. Our study demonstrates the TXNIP-mediated regulation of class I GLUTs, and sheds light on the potential role of oxidative stress in glucose metabolism. We identified three essential amino acid residues (K212, R238 and K287) within the C-terminal ARR which were critical for TXNIP-GLUT1 interaction. Upon interaction, TXNIP induced the downregulation of GLUT1 surface expression through its di-leucine motif, which triggered the lysosomal degradation of GLUT1. Evidently, the intermolecular disulfide bond formed between TXNIP and TRX dictated the availability of TXNIP for GLUT1 interaction since TXNIP robustly interacted with GLUT1 upon increases in intracellular ROS. These findings implicate the role of TXNIP in coordinating the crosstalk between oxidative stress and glucose transport.

The fact that both TXNIP and ARRDC4 are under the control of ChREBP/MondoA family of transcription factors and regulate class I GLUTs suggests that they are part of a larger regulatory network that controls glucose homeostasis [26–29]. Consistent with previous studies [7, 8, 30], our study also indicates that TXNIP and ARRDC4 function as part of a metabolic feedback loop to control glucose homeostasis as they readily downregulated GLUT-1, -3, and -4 and suppressed glucose uptake (Figs 2A–2E and 5). As observed by Patwari et al. [7], our findings also support the notion that the arrestin domain serves as a crucial structural element in the regulation of glucose metabolism. Specifically, the three positively charged residues (K212, R238, and K287) within the C-ARR domain of TXNIP served as a critical interface for GLUT1 interaction since mutation of these residues prevented TXNIP from interacting with GLUT1. Similarly, mutations of the charged residues, K243, T244, D290, and E308, at the C-ARR domain of ARRDC4 have also been shown to abolish GLUT1 interaction [30]. These indicate that the formation of a positively charged patch within the C-ARR domain is a conserved feature of TXNIP and ARRDC4 α-arrestins to control glucose homeostasis.

We and others have observed that ARRDC4 downregulates GLUT1 more efficiently than TXNIP, resulting in low glucose uptake [7, 30]. Interestingly, while TXNIP-3KA primarily displayed a nuclear localization (Fig 7D), ARRDC4-4K is present in both the cell membrane and cytoplasm [30]. This may explain more the potent effect on the degradation of class I GLUTs and the inhibition of glucose uptake by ARRDC4 compared to that by TXNIP (Fig 5A and 5B). Moreover, ARRDC4 does not interact with TRX, thereby escaping additional negative control [7], which may allow ARRDC4 to have a higher binding activity to GLUTs than TXNIP. These potential dissimilarities may explain differences in the regulation of class I GLUTs between TXNIP and ARRDC4.

Arrestins commonly serve as key mediators of plasma membrane protein homeostasis by remodeling the cell surface in response to environmental cues [31]. This feature is attributable to the conserved PY motif that utilizes the Endosomal Sorting Complex Required for Transport (ESCRT) pathway through interactions with the Homologous to E6AP C-terminus (HECT) ubiquitin ligases (WWP1, WWP2 and Itch) [32–34]. While the α-arrestins contain PY motifs at the C-terminus, only TXNIP contains an additional di-leucine motif. Furthermore, while ARRDC4 utilizes its PY motifs to downregulate GLUT1 through clathrin-mediated endocytosis [30], TXNIP uses its di-leucine motif to associate with clathrin and adaptor AP2 to trigger endocytosis [9]. Mutation of the PY motifs in TXNIP showed little to no effect on GLUT1 surface expression and degradation, while there was a marked decrease in endocytosis when the di-leucine motif was mutated. Thus, while the di-leucine motif of TXNIP is important for class I GLUTs downregulation, the C-terminal PY motifs may play roles in the regulation of other receptors or contribute to the downregulation of GLUTs under special conditions.

The co-localization of TXNIP-GLUT1 complexes within the early endosomes, late endosomes, and lysosomes provides evidence that these complexes were directly trafficked to the lysosomal pathway for degradation (Fig 4E). Furthermore, the inhibition of GLUT1 degradation by bafilomycin A1 suggested the lysosome-mediated downregulation of GLUT1 (Fig 4C). A recent time-laps confocal microscopy study also showed that TXNIP and clathrin simultaneously disappear from the plasma membrane [9]. Despite its strong GLUT1-binding affinity, the arrestin domain of TXNIP alone was not able to affect glucose uptake, suggesting that the TXNIP-mediated inhibition of glucose uptake is mainly due to the downregulation of GLUT1 surface expression. Similar to how the β-arrestins regulate GPCRs, where the β-arrestin is autoinhibited by its tightly bound C-terminal tail under basal state and needs a phosphorylated receptor and bound agonist to become efficiently active [35], it is possible that interaction of the C-ARR domain of TXNIP with GLUT1 may release its C-terminal di-leucine motif, which subsequently reveals itself to clathrin and AP-2 proteins to trigger endocytosis. Further detailed study is required to determine the timing and mechanism of TXNIP-mediated endocytosis and degradation of GLUT1.

TXNIP has been primarily studied for its interaction with TRX [11, 12, 24, 25]. TXNIP binds to the reduced form of TRX through an intermolecular disulfide bond, which inhibits TRX’s reducing activity. Particularly, our previous crystal structure of the TRX–TXNIP complex has shown that upon binding to TRX, TXNIP undergoes a structural rearrangement that involves the switching of a head-to-tail interprotomer C63-C247 disulfide bond between TXNIP molecules to an interdomain C63-C190 disulfide bond, and the formation of a *de novo* intermolecular disulfide bond between TXNIP C247 and TRX C32 [24]. Patwari et al. [7] have shown that TXNIP C247 mutation prevents the association with TRX but still reduces glucose uptake in mature adipocytes and in primary skin fibroblasts, suggesting that TXNIP controls glucose metabolism independently of its interaction with TRX. Consistently, we also showed that TXNIP-C247S displayed GLUT1 binding (Fig 6B) and suppressed glucose uptake as efficiently as TXNIP-WT (Fig 7F and 7G), and that expression of TXNIP-WT or TXNIP-C247S led to similar effects on glucose metabolism (Fig 7H and S6A-S6D Fig in S1 File). Strikingly, upon H_2_O_2_ treatment, a powerful oxidizing agent, TXNIP strongly interacted with GLUT1 (Fig 6I), suggesting that the intermolecular disulfide bond formed between TXNIP and TRX dictates the availability of TXNIP to interact with GLUT1. This implicates TXNIP as an important linker between oxidative stress and glucose metabolism.

As glucose is the fundamental metabolite in all organisms, the uptake of glucose has to be tightly and precisely regulated for homeostasis and metabolic harmony (S7 Fig in S1 File). Under normal conditions, TXNIP functions as a gatekeeper of TRX by default. Upon high glucose intake or acute extracellular stimuli, intracellular ROS levels rise and interrupt the disulfide bond between TRX-TXNIP. Subsequently, TXNIP binds to and degrades class I GLUTs, reducing glucose influx and thereby returning intracellular ROS to basal levels. Thus, TXNIP shuffles between TRX and GLUT to orchestrate the intracellular redox potential and glucose transport, suggesting that TXNIP is a promising therapeutic target for metabolic diseases.

## Material and methods

### Animal care

TXNIP+/+ and TXNIP-/- C56/BL6 mice were bred and housed in specific pathogen free (SPF) facilities with controlled temperature (20–26°C), illumination (10 h light/14 h dark), and humidity (30–70%) at the University of Southern California Animal Research Center at Keck Medical School and Lerner Research Institute Biological Resources Unit at Cleveland Clinic. TXNIP+/+ and TXNIP-/- mice were anesthetized with isoflurane and organs were harvested for immunohistochemistry and immunoblotting. All mice studies were carried out in strict accordance to the protocols reviewed and approved by the Institutional Animal Care and Use Committees (IACUC) at the University of Southern California Keck Medical School (Protocol Number: 11842 and 20052) and the Cleveland Clinic (Protocol Number: 2481).

### Tissue culture

HEK293T cells and HeLa cells were purchased from American Type Culture Collection (ATCC) and maintained in Dulbecco’s Modified Eagle’s Medium (DMEM; Gibco) containing 4 mM glutamine, 4.5 g/L glucose and 10% FBS, and 1% Penicillin-streptomycin. Transient transfections were performed with Lipofectamine 2000 (Invitrogen) according to the manufacturer’s instructions. HEK293T and HeLa stable cell lines were generated using a standard selection protocol with puromycin or hygromycin.

### Western blots and immuno-precipitation

Cells were washed three times with cold PBS and lysed in 1% NP40 lysis buffer (20 mM Tris7.5, 150 mM NaCl, 1% SDS, 1% deoxycholate, and protease inhibitor cocktail). Lysate was spun for 15 min at 13000 rpm in 4°C, and the supernatant was resolved on SDS-PAGE for western blot or precleared for immune-precipitation. Primary antibody was added to the supernatant and incubated overnight at 4°C. Immune complexes were captured by incubation with protein A/G resin for another 1 hr at 4°C, then washed 3 times with lysis buffer, and finally mixed with 2X sample buffer for western blot analysis.

### Lentivirus package

HEK293T cells were transfected with pCDH plasmid encoding indicated protein together with packing plasmids: Gag/Pol, Rev, and VSV-G. At 8 hrs after transfection, the culture medium was replaced. The supernatant was collected 48 hrs post transfection and passed through a 0.45 μm filter.

### ^3^H-2DG uptake

HeLa cells in 12-well tissue culture plates were incubated with DMEM medium for 2 hrs, followed by glucose-free DMEM incubation for 30 min. 100 μM 2-deoxyglucose and [^3^H]-2-deoxyglucose (0.1 mCi/well) were added to the medium and incubated for another 10 min before washing with cold PBS twice. 400 ul of 1% SDS was added to lyse the cell for 10 min at room temperature and the lysate was collected and mixed with 10ml Scintillation Cocktails (Perkin Elmer #6013681). The mixture was read using liquid scintillation counter LS6500 (Beckman Coulter).

### Immunohistochemistry

The paraffin blocks of mice tissues were sectioned at 5 μm thickness and stained with TXNIP, GLUT1, or GLUT3 antibody. HRP-linked Anti-rabbit IgG Antibody, Donkey Anti-Rabbit IgG (H+L) Secondary Antibody, and Alexa Fluor 488 were used for detection.

### Confocal fluorescence microscopy

For immunostaining, HEK293T or Hela were seeded on 24-well culture dishes that contained 12 mm diameter round glass coverslips. 48 hrs after transfections, cells were fixed with 4% paraformaldehyde in PBS at 4°C for 10 min and permeabilized with 0.25% Triton X-100 in PBS for 15 min before being blocked with 5% BSA for 1 hr at room temperature. Slides were then stained with respective primary antibodies overnight at 4°C. After washing to remove excess primary antibodies, the slides were incubated for 1 hr at room temperature with fluorescently labeled secondary antibodies (Molecular Probes). The coverslips were washed 3 times with PBS and mounted on slides with mounting media (Biotium). BiFC assay with CellMask were performed in 6-well dishes containing glass bottoms. Cells were stained with CellMask according to the manufacturer’s instructions and were fixed with 4% PFA prior to imaging. Slides were imaged on a Keyence bz-x700 microscope or Nikon Eclipse C1 laser-scanning confocal microscope and 6-well dishes were imaged on a Leica SP8 confocal microscope.

### Metabolomics with LC-MS and data processing

Liquid Chromatography-Mass Spectrometry (LC-MS)–based metabolomics analysis was used to report alternate metabolic state of cells expressing various states of TXNIP. At 48 hrs after lentivirus infection, cells were washed with 4°C PBS 3 times, then immersed in 2 ml of ice-cold 80% methanol (LCMS grade), and finally incubated for at least 3 hrs in -80°C. All cells were detached from the plates and transferred to 2 ml screw cap tube. The cells were centrifuged at 12,000 rpm for 10 min in 4°C. Next, the supernatant was transferred to fresh 2 ml screw cap tube, and it was dried down completely. The dried pellet was re-suspended in 200 μl of pre-cooled acetonitrile/methanol/H_2_O (2:2:1) and applied to LC-MS after removing undissolved pellets by centrifugation.

Metabolite differentiation and detection were performed with an Agilent Accurate Mass 6230 TOF coupled with an Agilent 1290 Liquid Chromatography system using a Cogent Diamond Hydride Type C column (Microsolve Technologies) as described previously [36]. Briefly, the mobile phase consisted of the following: solvent A (ddH_2_O with 0.2% formic acid) and solvent B (acetonitrile with 0.2% formic acid). The gradient used was as follows: 0–2 min, 85% B; 3–5 min, 80% B; 6–7 min, 75%; 8–9 min, 70% B; 10–11.1 min, 50% B; 11.1–14 min 20% B; 14.1–24 min 5% B followed by a 10 min re-equilibration period at 85% B at a flow rate of 0.4 ml/min. Mass axis dynamics was calibrated by continuous infusion of a reference mass solution using an isocratic pump. This configuration achieved mass errors of 5 ppm, mass resolution ranging from 10,000 to 25,000 (over m/z 121–955 atomic mass units), and 5 log_10_ dynamic range. Metabolite identities were searched for using a mass tolerance of <0.005 Da. Metabolite concentrations were normalized to protein concentration based on measurement in individual samples using the Pierce BCA Protein Assay Kit. Data were analyzed using Profinder B.06.00 software (Agilent Technologies). Heatmaps and Principal Component Analysis (PCA) were using the web-based program, MetaboAnalyst 5.0 (https://www.metaboanalyst.ca/MetaboAnalyst/home.xhtml).

### Quantification of surface HA-GLUT1

HeLa cells stably expressing HA-GLUT1 were infected with lentivirus for 12 hrs. Fresh complete culture medium was applied for another 36 hrs. Cells were washed with PBS twice, treated with trypsin for 20 sec, and then collected with fresh medium. The cells were fixed with 4% paraformaldehyde for 10 min and blocked with PBS containing 5% BSA for another 30 min. Cells were incubated with anti-HA (1:500) for 1hr at room temperature. After washing, the cells were incubated with Alexa Fluor 488 labelled secondary antibody (1:1000) for 40 min. After washing, the cells were resuspended in FACS buffer (5% FBS in PBS) and analyzed by Attune NxT flow cytometer (ThermoFisher Scientific). FCS data were analyzed using FlowJo software.

### Statistical analysis

All data are expressed as Mean ± SEM, unless otherwise noted. Statistical significance across two groups were tested by Student’s t-test.

## Supporting information

S1 File Contains all supporting figures. (PDF)

S1 Raw images (PDF)
