# Supplementary material for: TXNIP-mediated crosstalk between oxidative stress and glucose metabolism
Source: PLoS One. 2024 Feb 8;19(2):e0292655. doi: 10.1371/journal.pone.0292655 (PMC10852281; doi:10.1371/journal.pone.0292655)
Supplement: S1 File — (PDF) [file pone.0292655.s001.pdf]

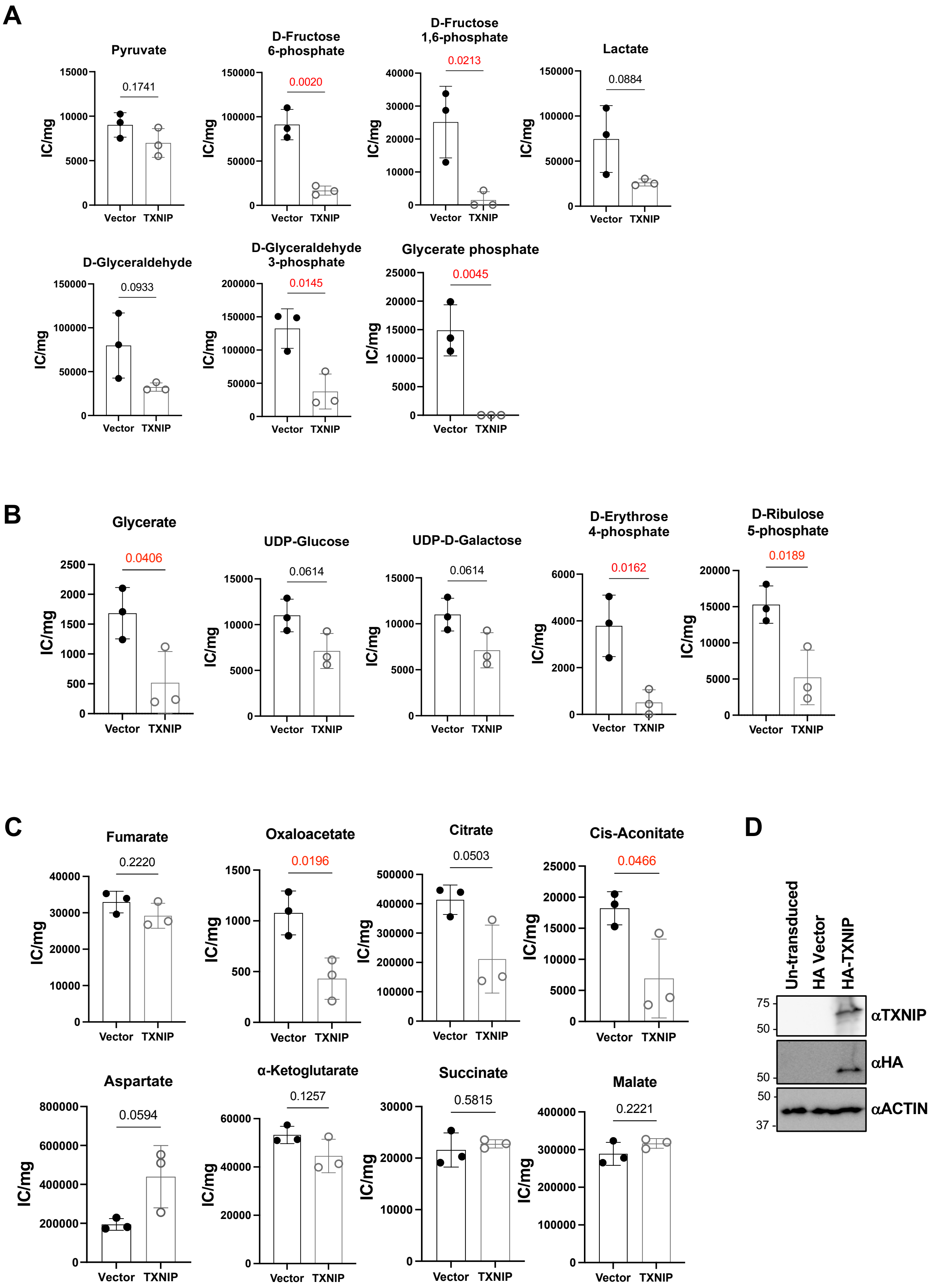

Figure S1. The effect of TXNIP on glucose metabolism in HEK293T cells

A

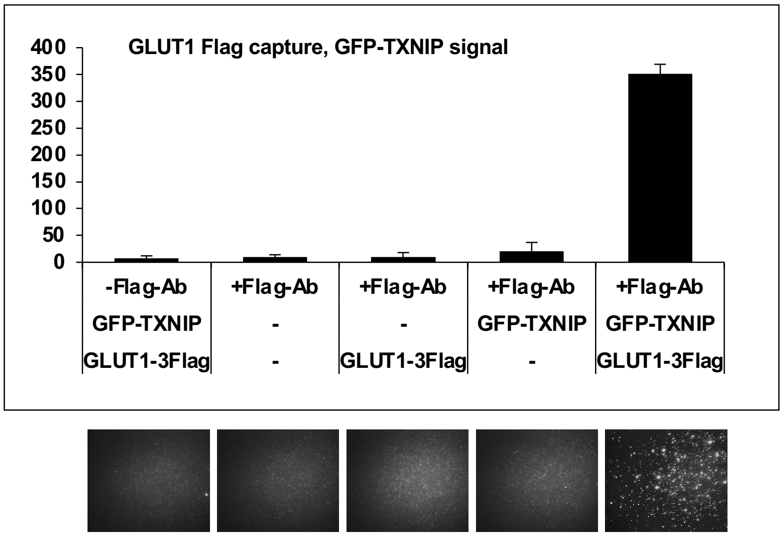

B

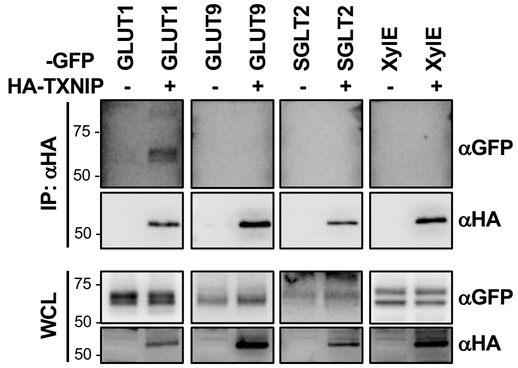

Figure S2. TXNIP specifically interacts with class I GLUTs

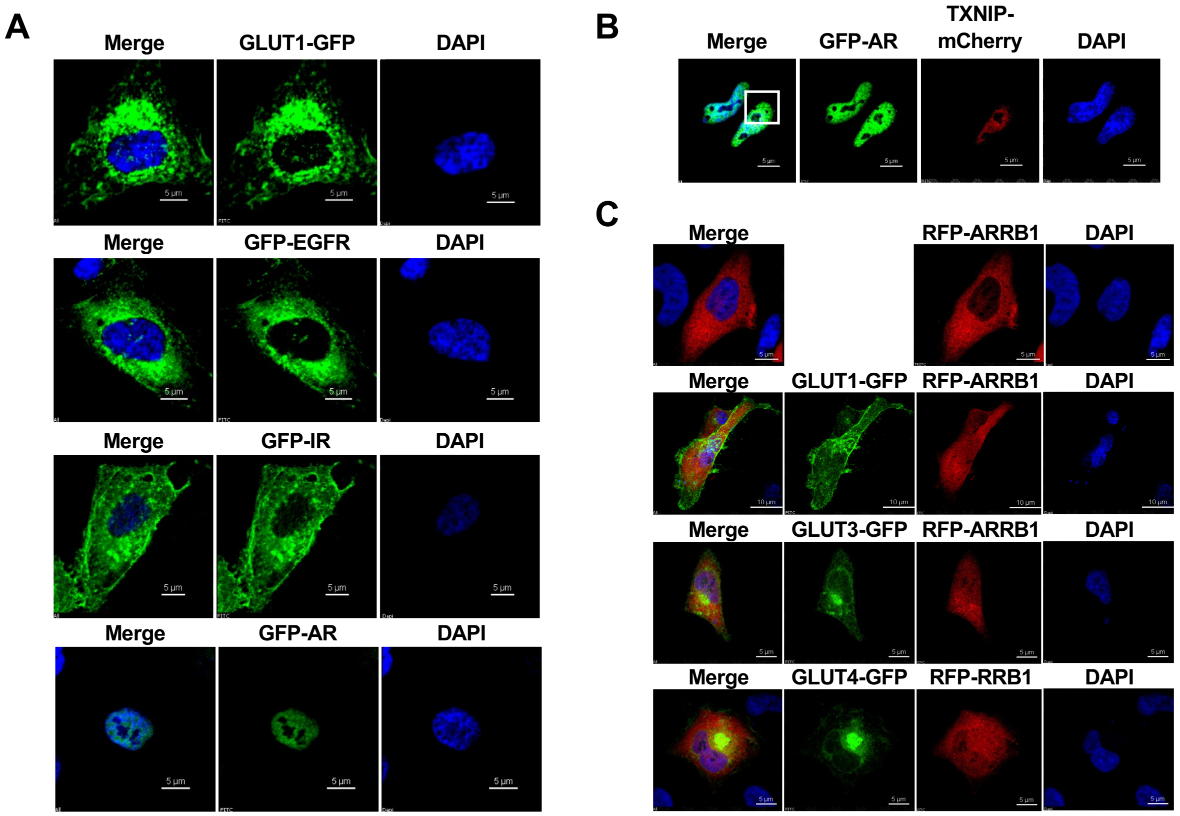

Figure S3. TXNIP interacts with class I GLUTs on the membrane through its central arrestin domain

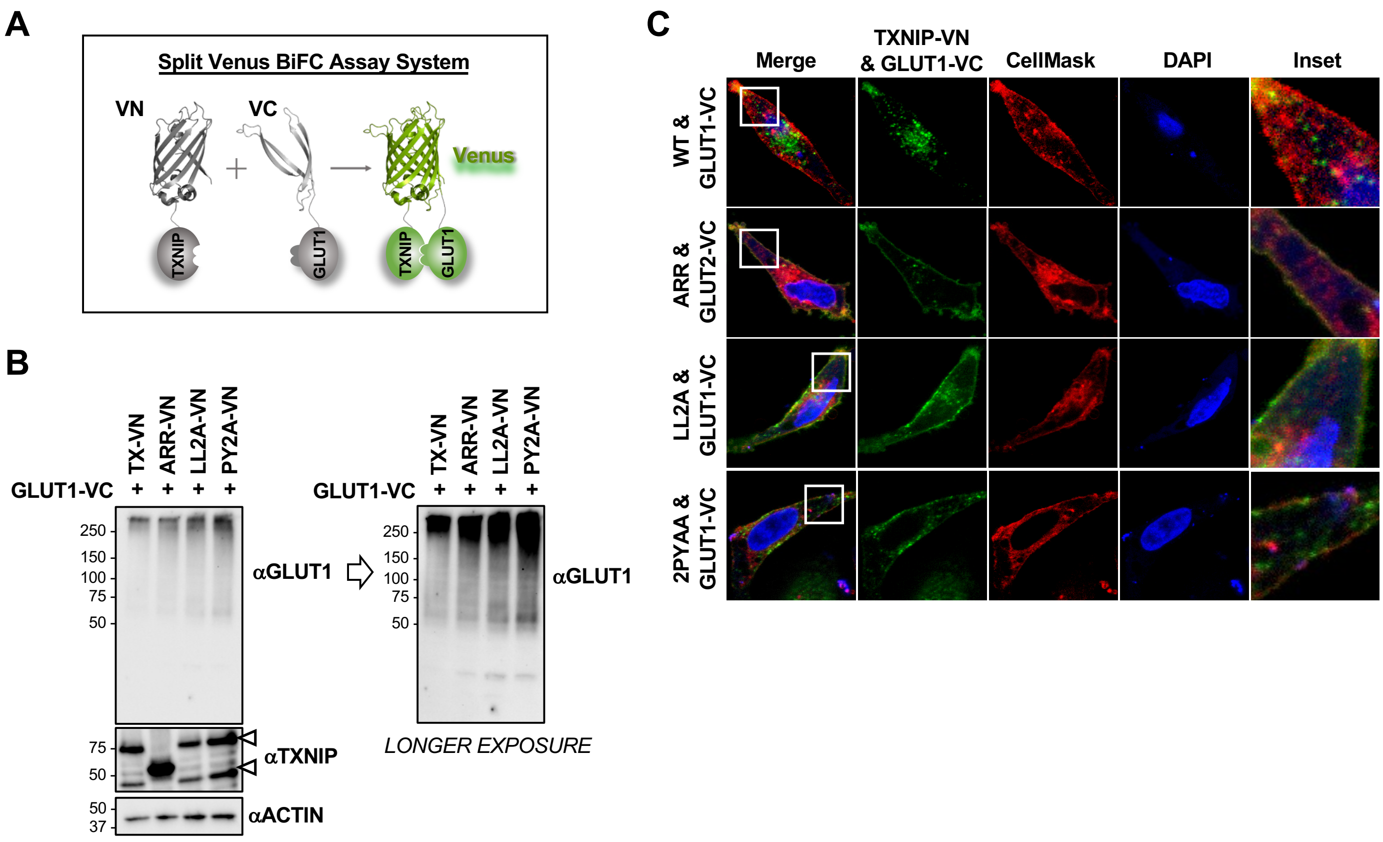

Figure S4. TXNIP interaction mediates the lysosomal degradation of GLUT1

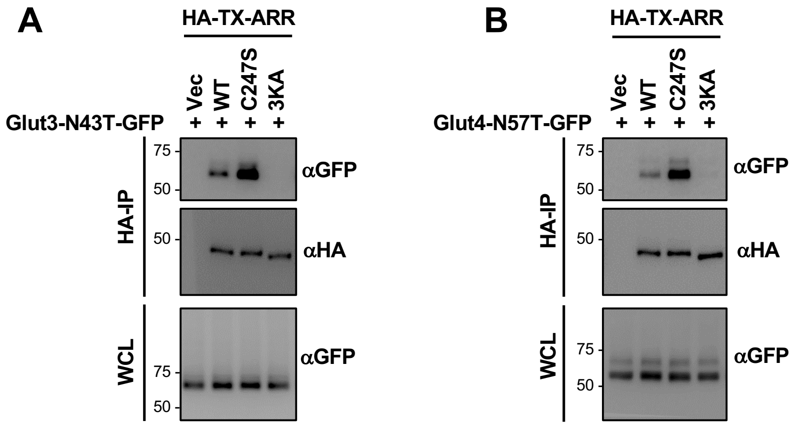

Figure S5. Class I GLUTs share similar binding features to TXNIP

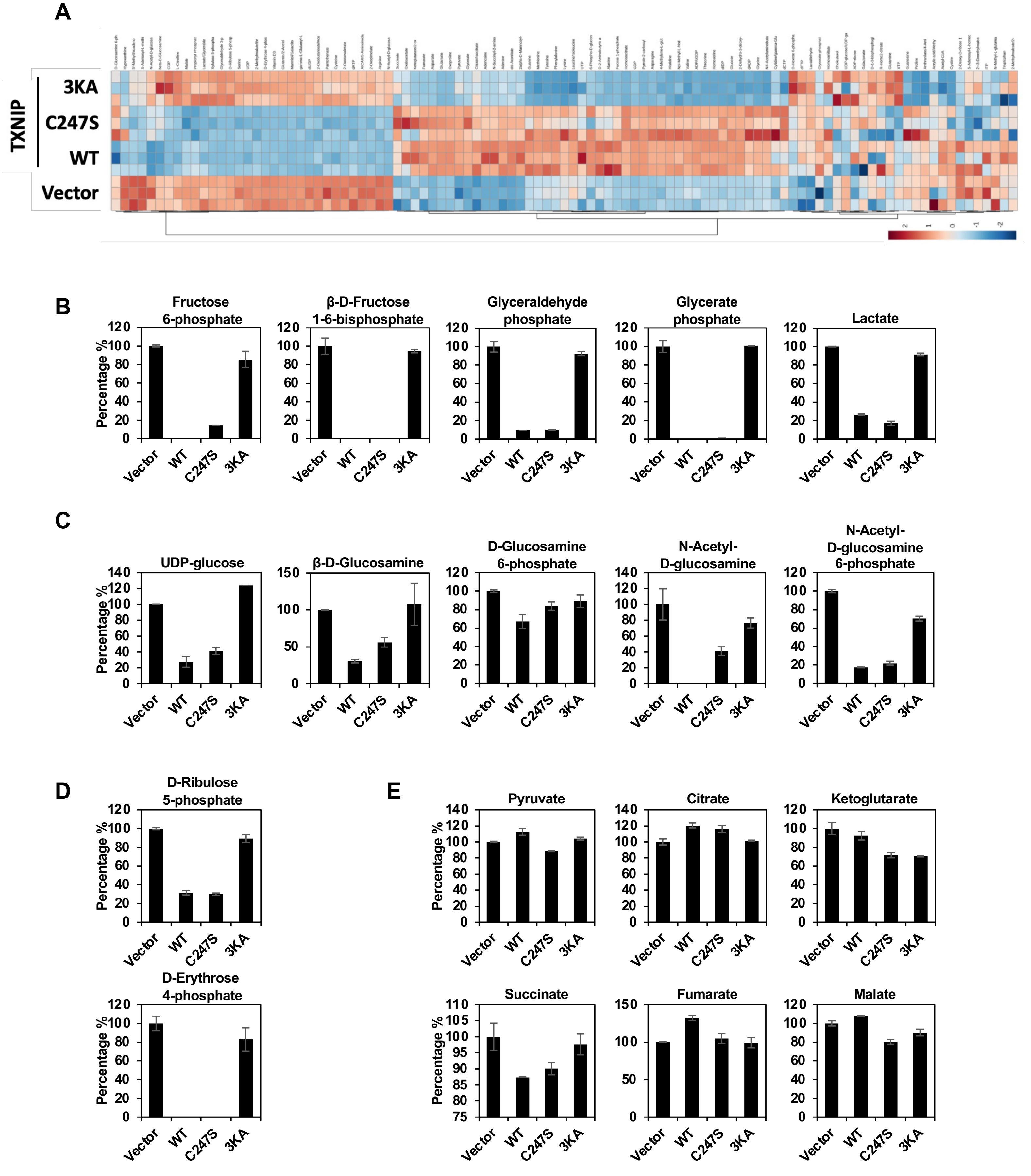

Figure S6. The positively charged interface of TXNIP central arrestin domain is essential for GLUT1 interaction and glucose metabolism

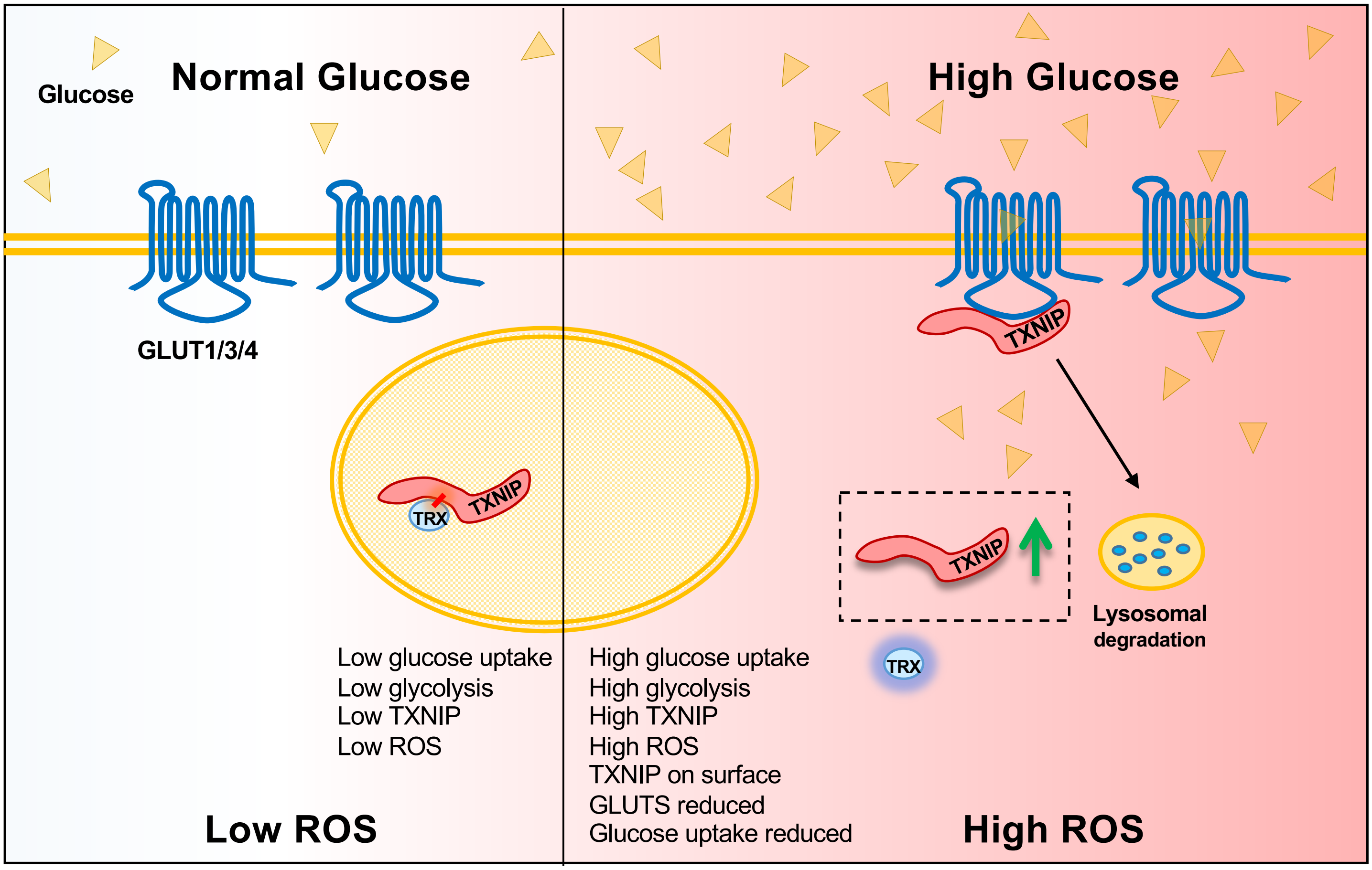

Figure S7. Proposed model of TXNIP-mediated crosstalk between oxidative stress and glucose metabolism
