# Supplementary material for: TXNIP-mediated crosstalk between oxidative stress and glucose metabolism
Source: PLoS One. 2024 Feb 8;19(2):e0292655. doi: 10.1371/journal.pone.0292655 (PMC10852281; doi:10.1371/journal.pone.0292655)
Supplement: S1 Raw images — (PDF) [file pone.0292655.s002.pdf]

2A

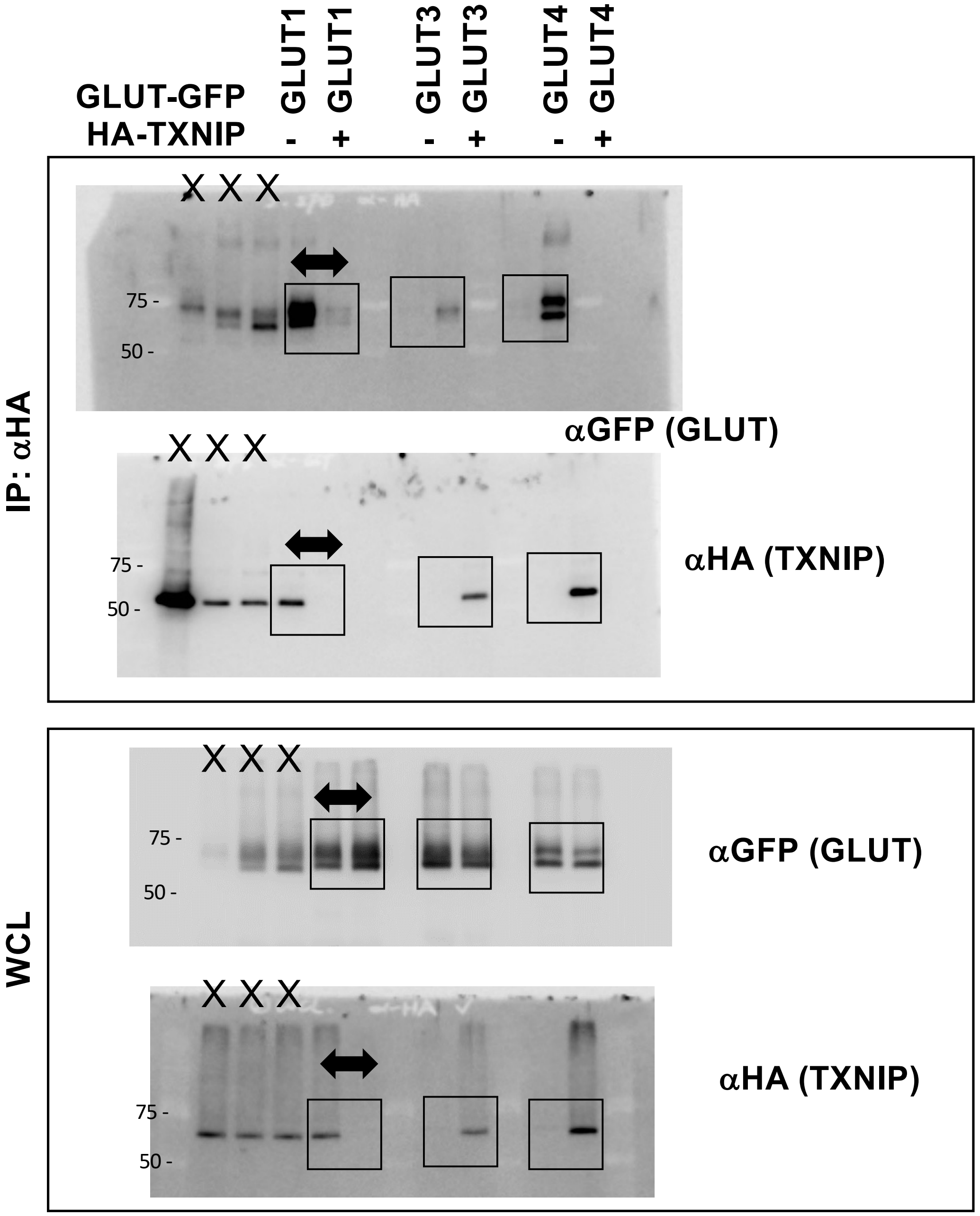
GLUT-GFP
HA-TXNIP
GLUT1 GLUT1 GLUT3 GLUT3 GLUT4 GLUT4
- + - + - +
IP: αHA
75 -
50 -
αGFP (GLUT)
αHA (TXNIP)
WCL
αGFP (GLUT)
αHA (TXNIP)

2C

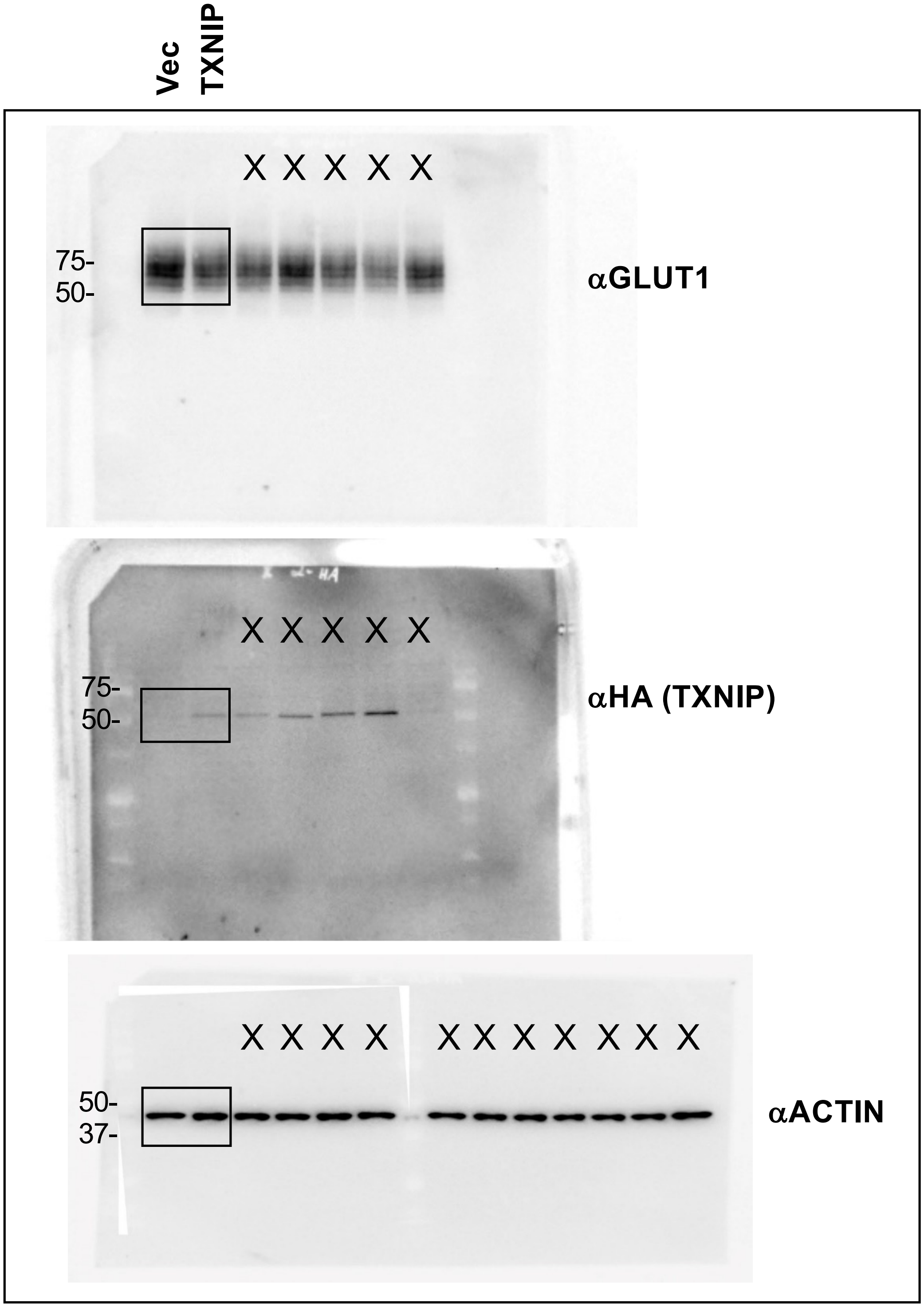
Vec
TXNIP
X X X X X
75-
50-
αGLUT1
X X X X X
75-
50-
αHA (TXNIP)
X X X X
X X X X X X X
50-
37-
αACTIN

2H

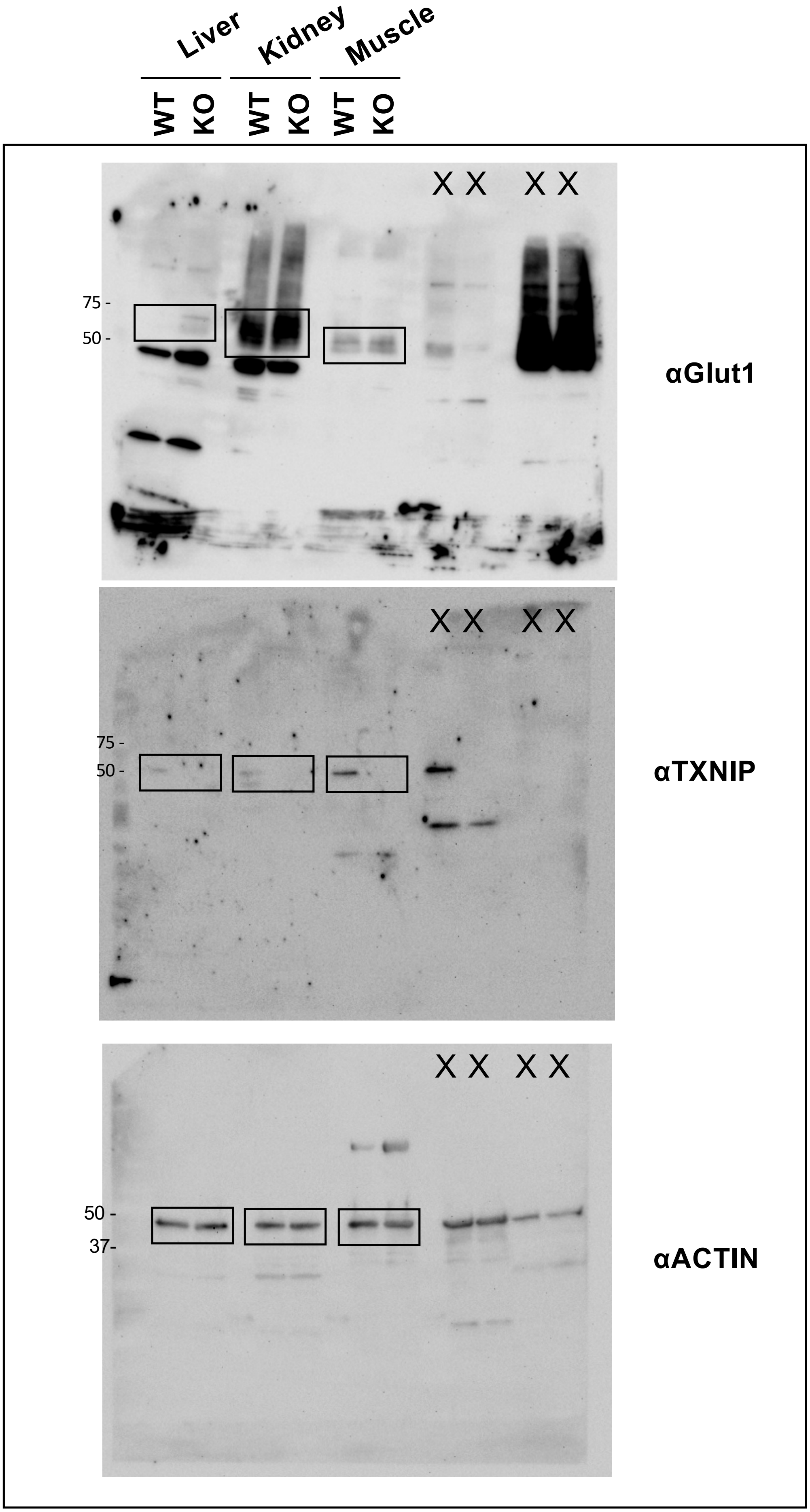

Liver
Kidney
Muscle
WT
KO
WT
KO
WT
KO
X X X X
75 -
50 -
αGlut1
X X X X
75 -
50 -
αTXNIP
X X X X
50 -
37-
αACTIN

2I

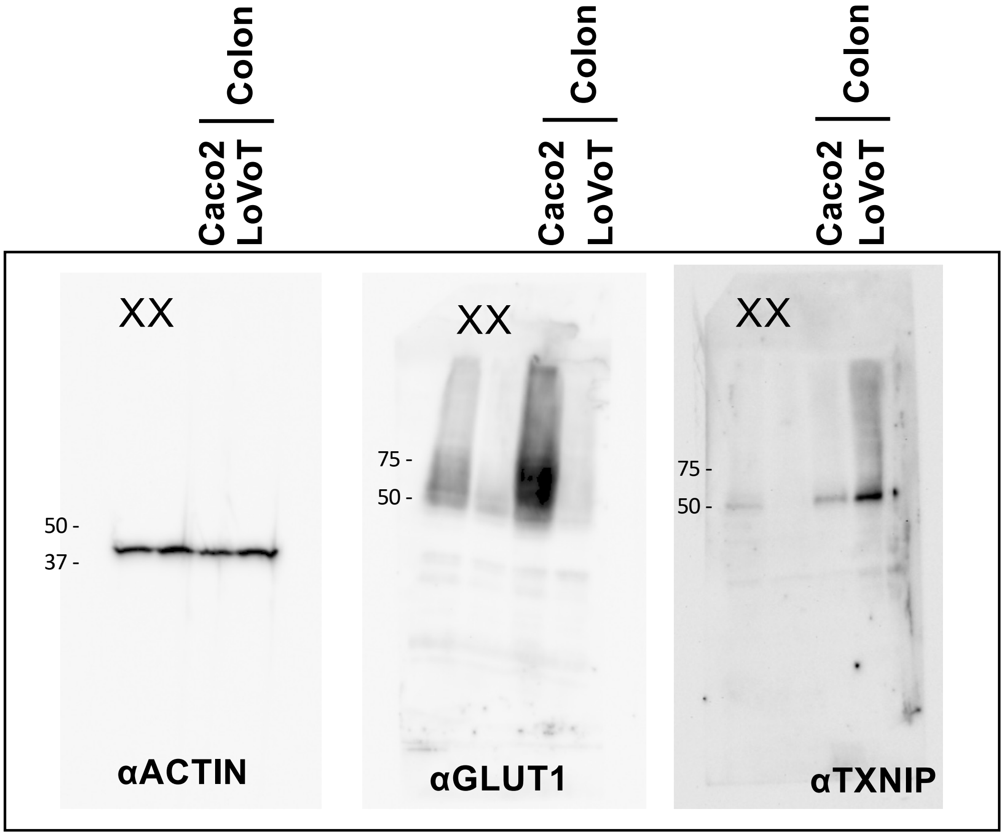

3F

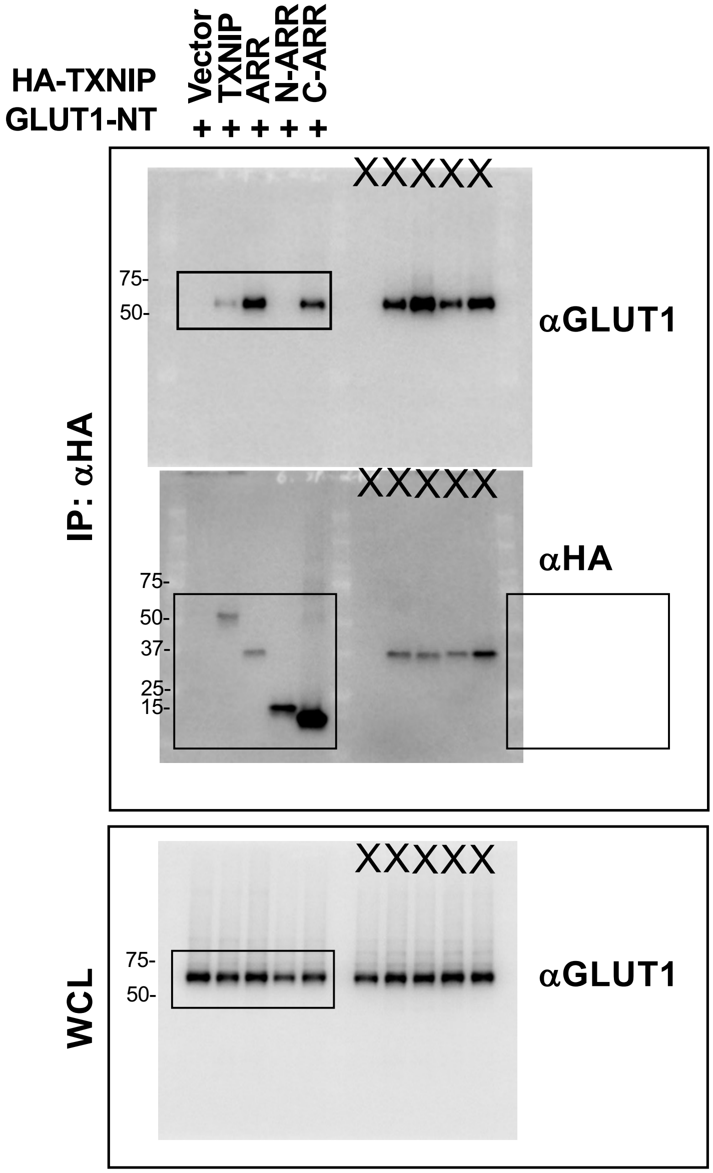
HA-TXNIP
GLUT1-NT
Vector
TXNIP
ARR
N-ARR
C-ARR
+ + + + +
XXXXX
75-
50-
αGLUT1
IP: αHA
XXXXX
αHA
75-
50-
37-
25-
15-
WCL
XXXXX
75-
50-
αGLUT1

4C

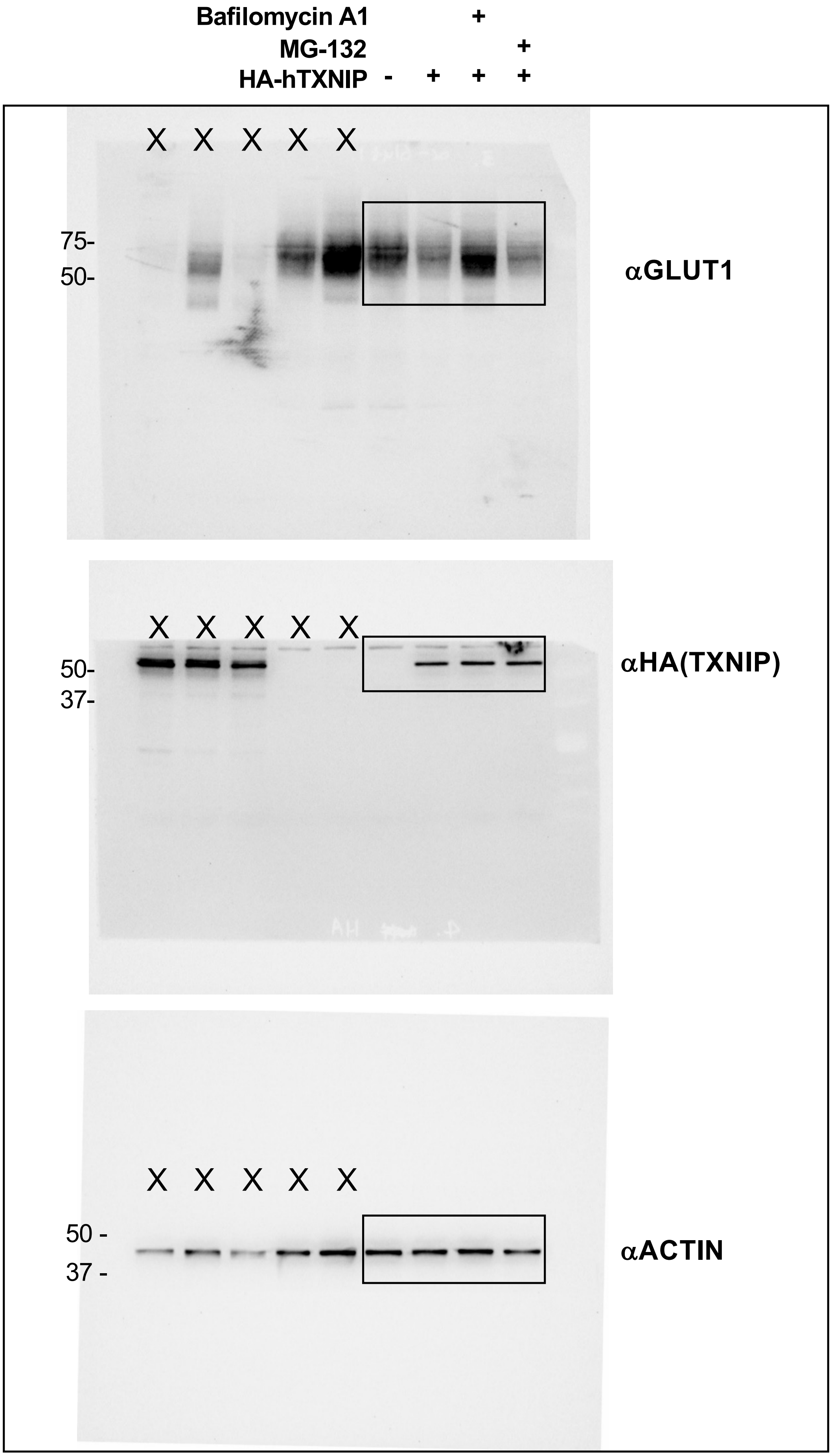

Bafilomycin A1 +
MG-132 +
HA-hTXNIP - + + +
X X X X X
75-
50-
αGLUT1
X X X X X
50-
37-
αHA(TXNIP)
X X X X X
50 -
37 -
αACTIN

4G

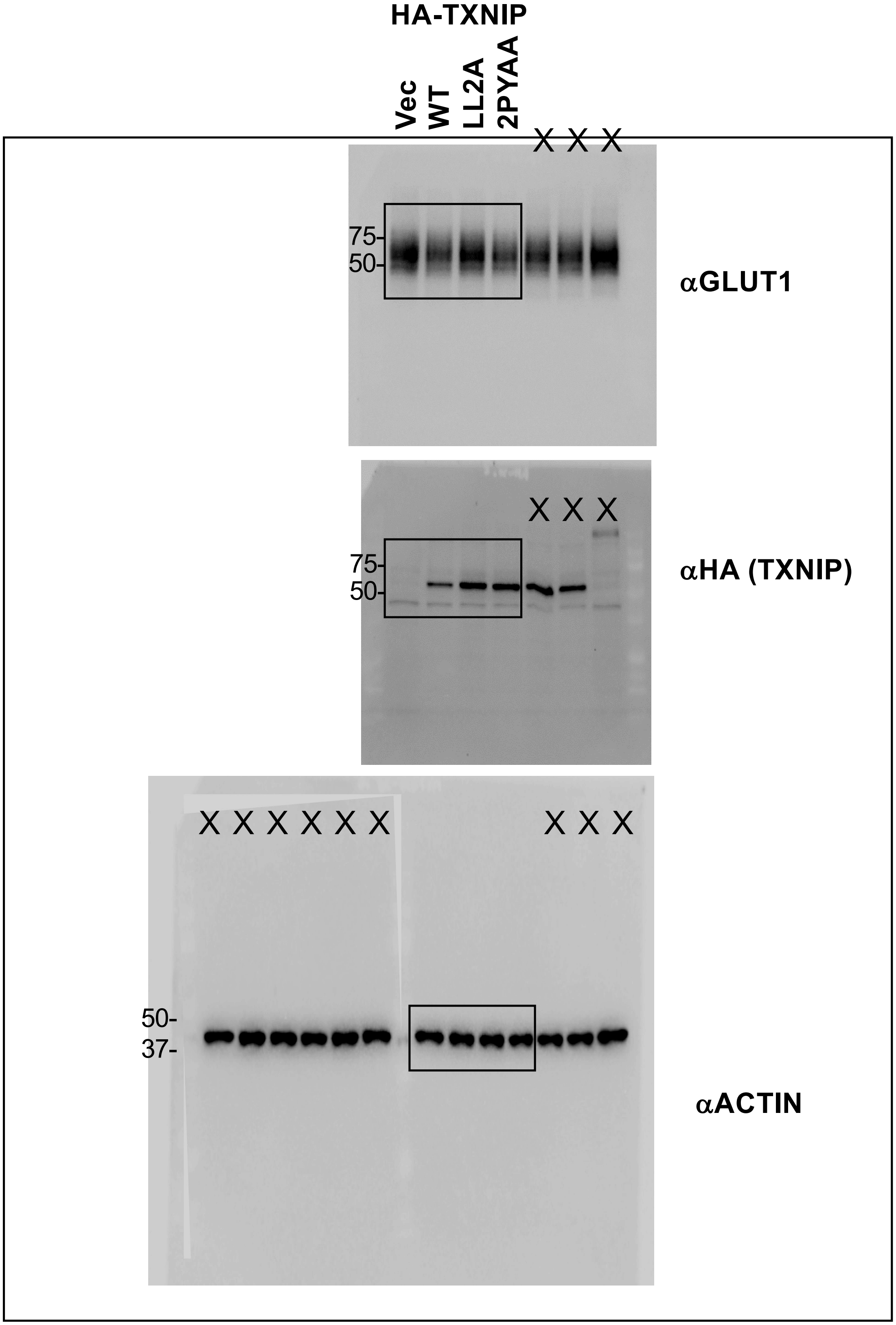
HA-TXNIP
Vec
WT
LL2A
2PYAA
X X X
75-
50-
αGLUT1
X X X
75-
50-
αHA (TXNIP)
X X X X X X
X X X
50-
37-
αACTIN

5C

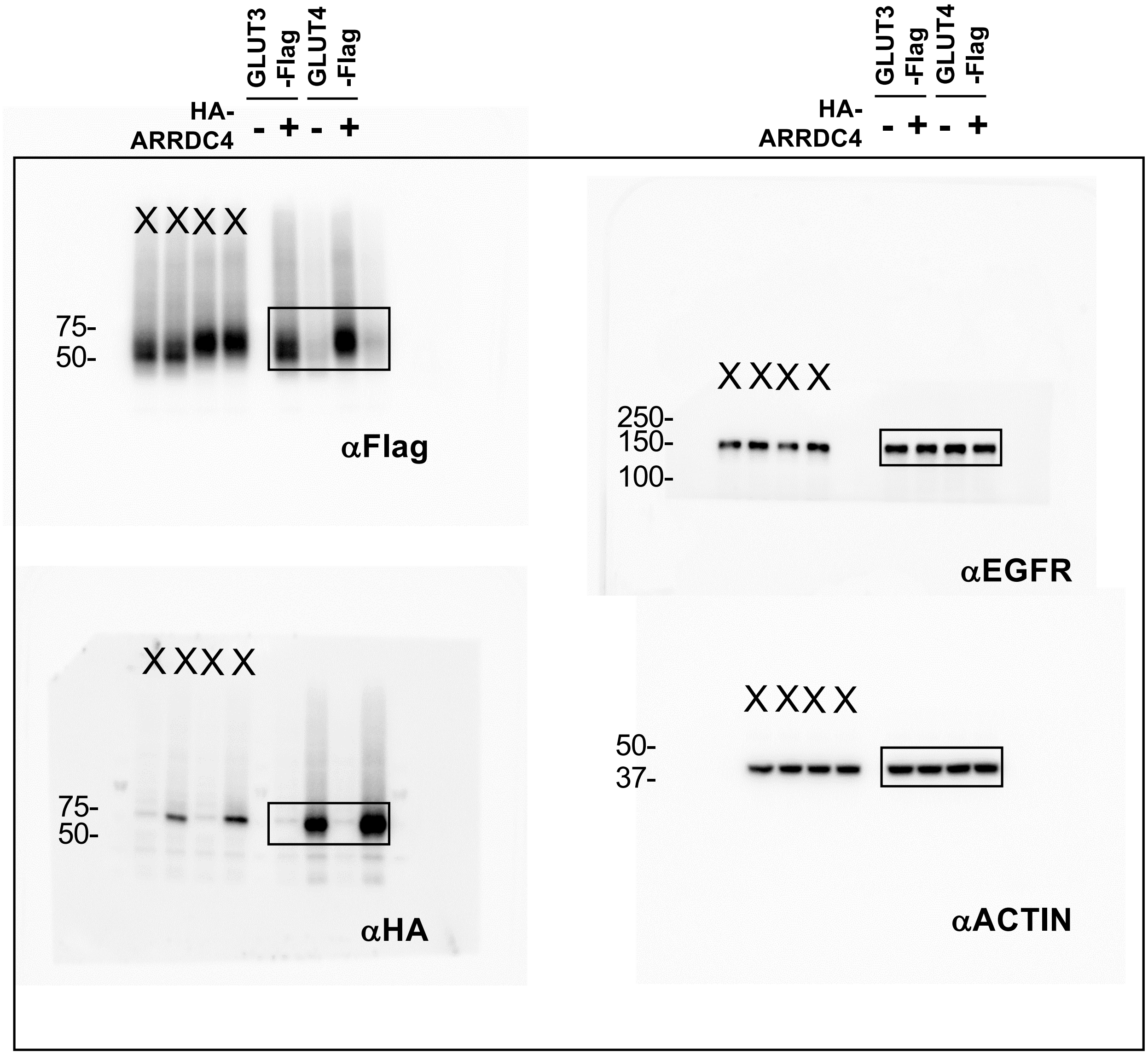

GLUT3 -Flag
GLUT4 -Flag
HA-ARRDC4
- + - +
75-
50-
αFlag
250-
150-
100-
αEGFR
75-
50-
αHA
50-
37-
αACTIN

6B

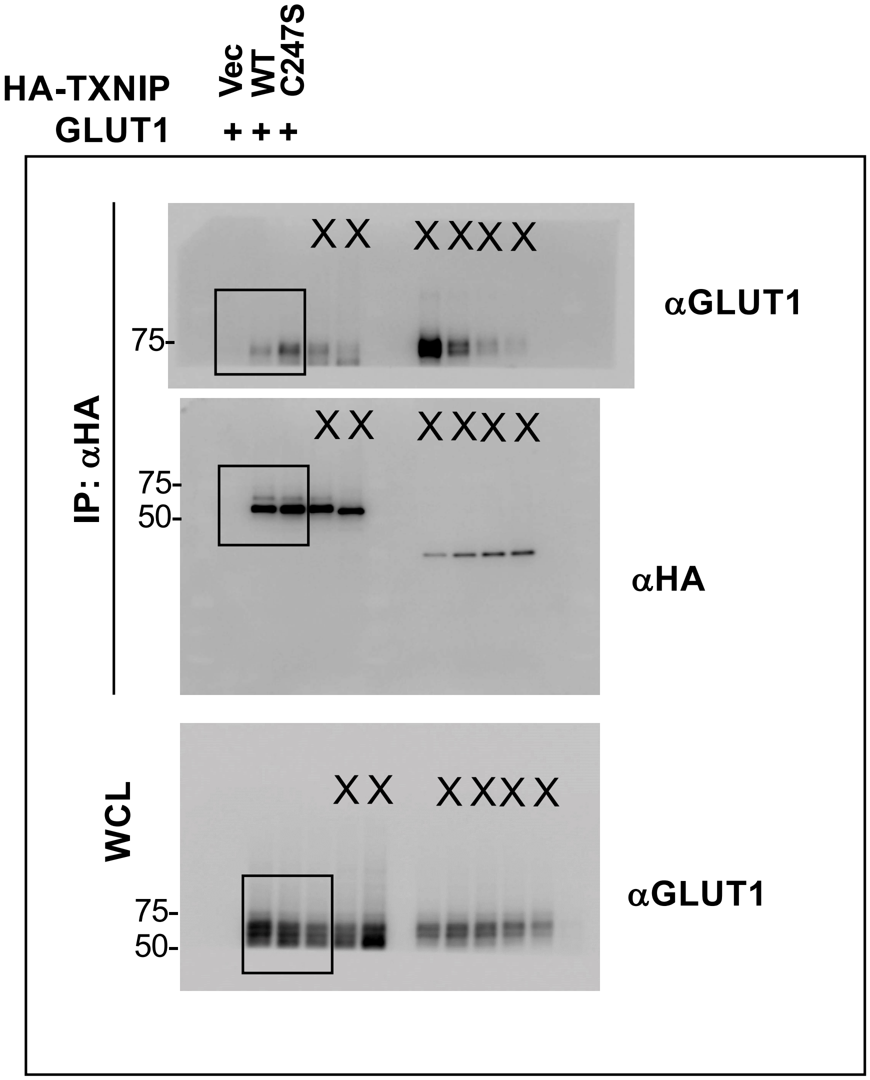
HA-TXNIP
GLUT1
Vec
WT
C247S
+++
XX XXXX
αGLUT1
75-
IP: αHA
XX XXXX
75-
50-
αHA
WCL
XX XXXX
75-
50-
αGLUT1

6C

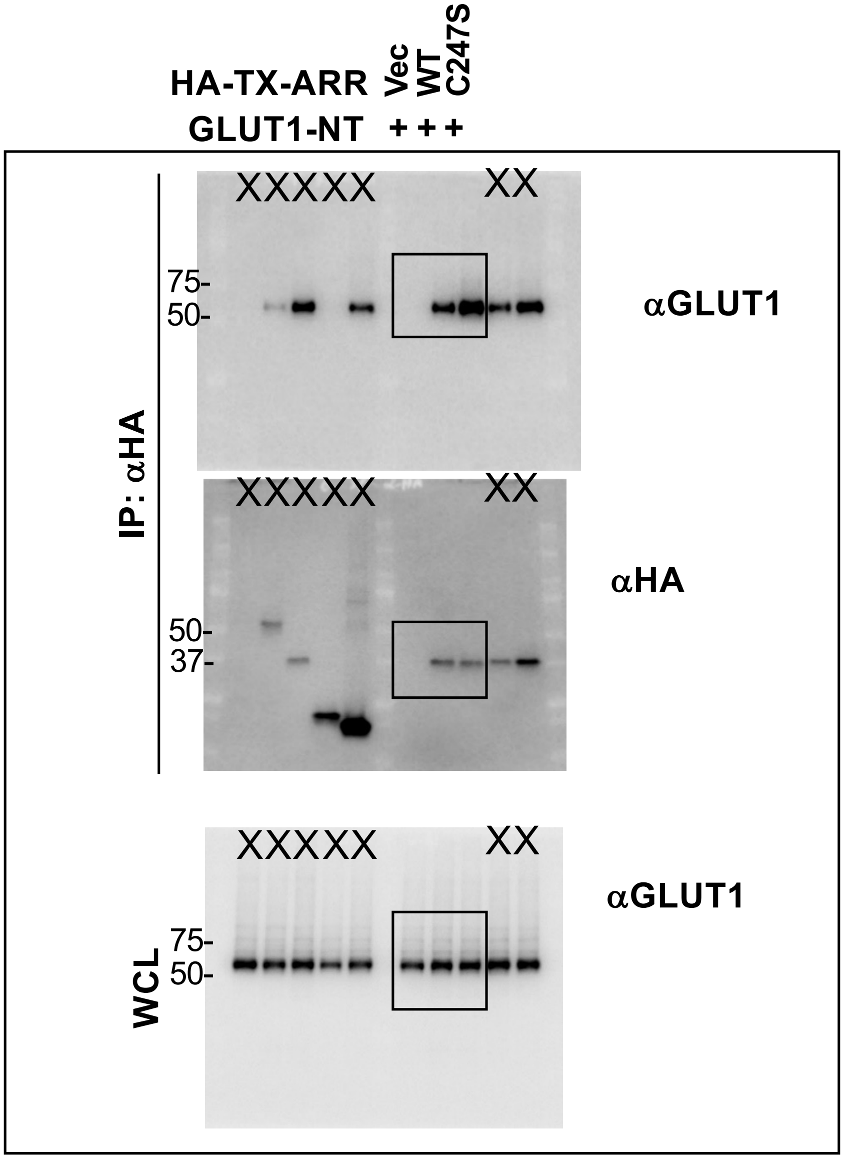
HA-TX-ARR
Vec
WT
C247S
GLUT1-NT
+ + +
XXXXX
XX
75-
50-
αGLUT1
IP: αHA
50-
37-
αHA
WCL
75-
50-
αGLUT1

6D

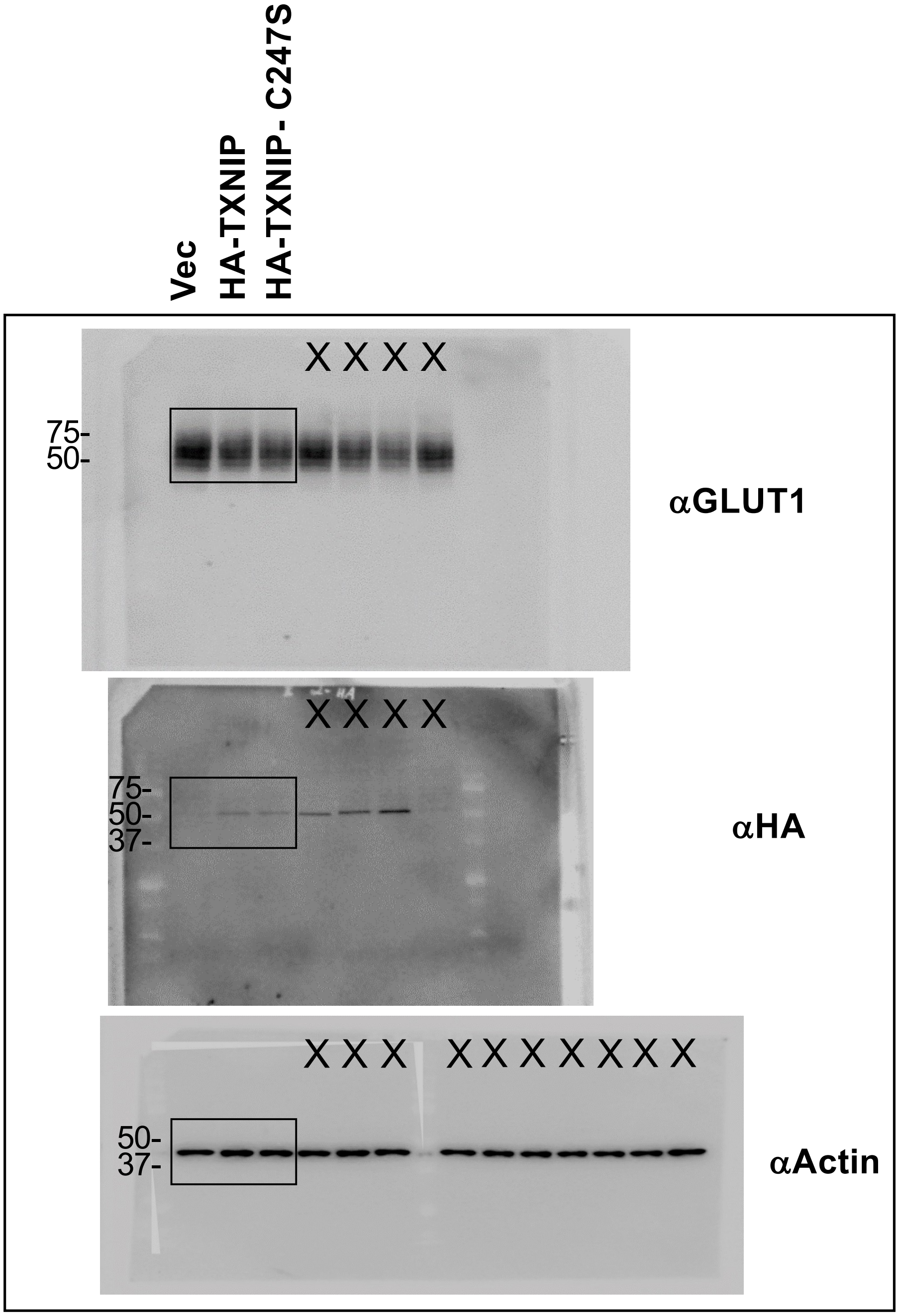
Vec
HA-TXNIP
HA-TXNIP- C247S
X X X X
75-
50-
αGLUT1
X X X X
75-
50-
37-
αHA
X X X
X X X X X X X
50-
37-
αActin

6G

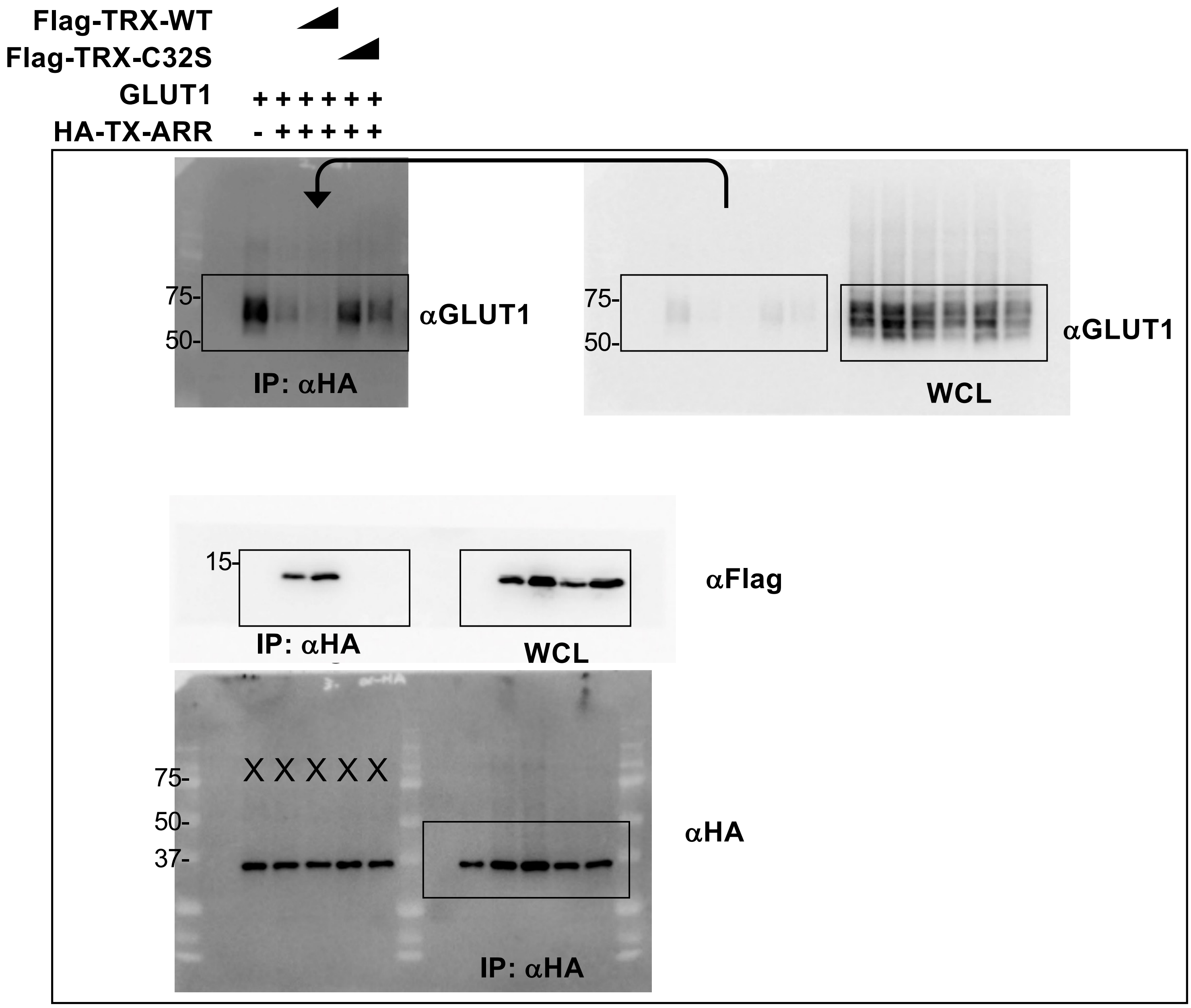

Flag-TRX-WT
Flag-TRX-C32S
GLUT1 + + + + + + +
HA-TX-ARR - + + + + + +
75-
50-
αGLUT1
IP: αHA
75-
50-
αGLUT1
WCL
15-
αFlag
IP: αHA
WCL
X X X X X
75-
50-
37-
αHA
IP: αHA

6H

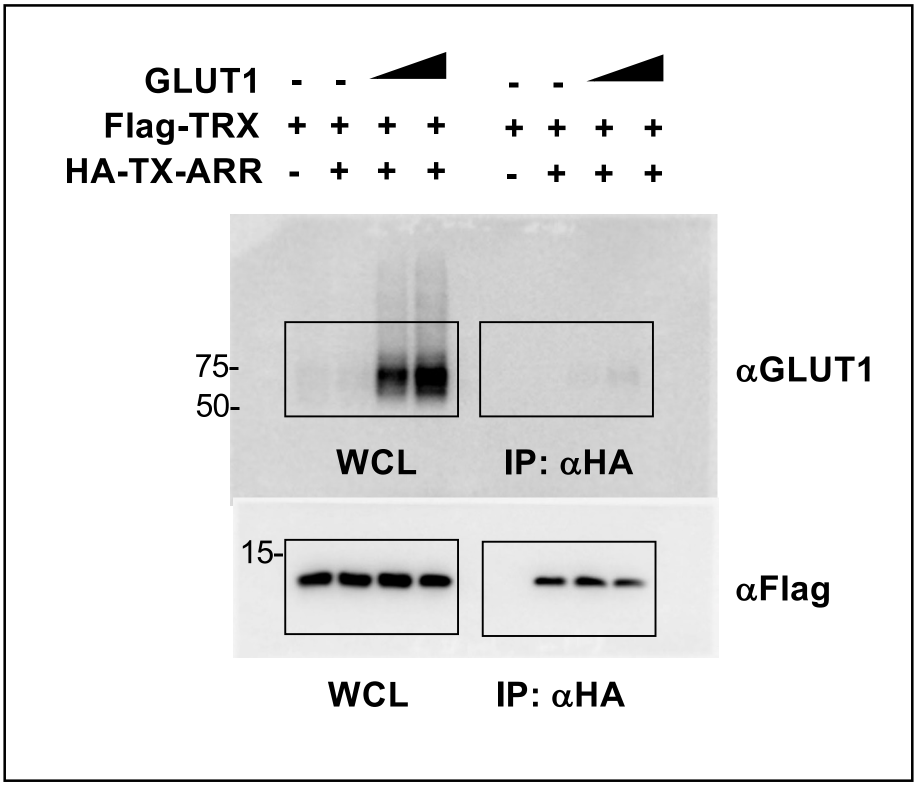
GLUT1 - -
Flag-TRX + + + +
HA-TX-ARR - + + +
75-
50-
WCL
IP: αHA
αGLUT1
15-
αFlag
WCL
IP: αHA

6I

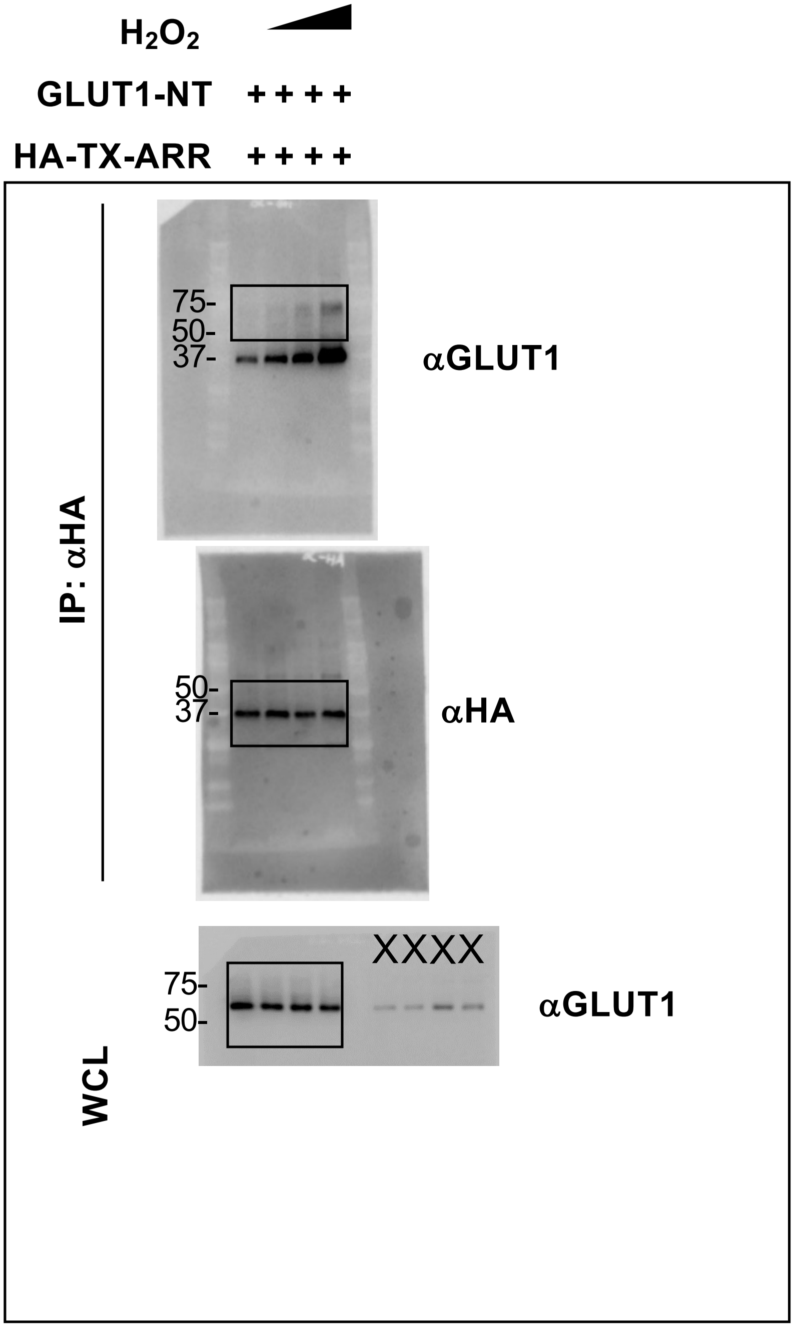

H_2O_2
GLUT1-NT + + + +
HA-TX-ARR + + + +
IP: αHA
75-
50-
37-
αGLUT1
50-
37-
αHA
WCL
XXXX
75-
50-
αGLUT1

6J

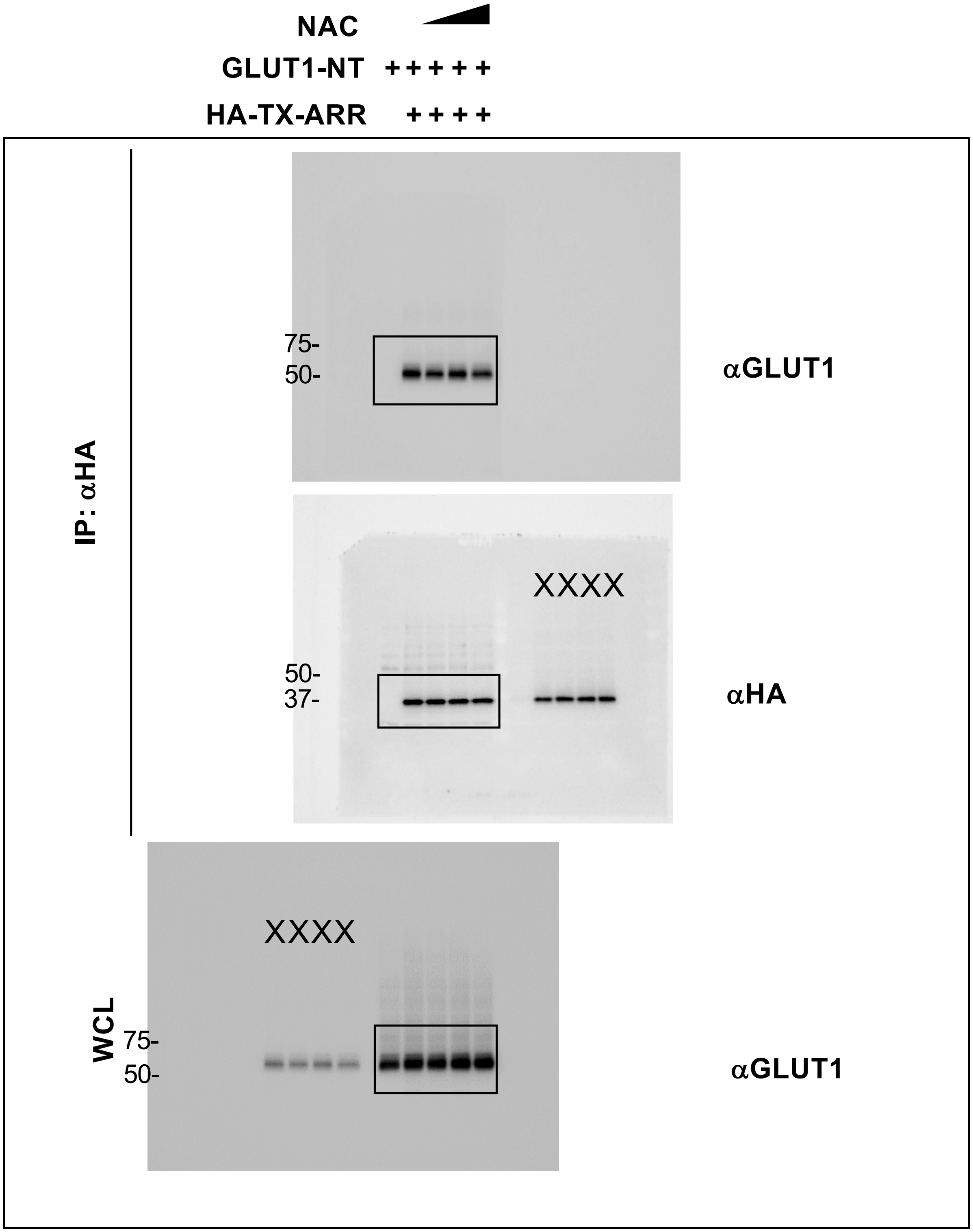

NAC
GLUT1-NT + + + + + +
HA-TX-ARR + + + + +
IP: αHA
75-
50-
αGLUT1
XXXX
50-
37-
αHA
WCL
XXXX
75-
50-
αGLUT1

7B

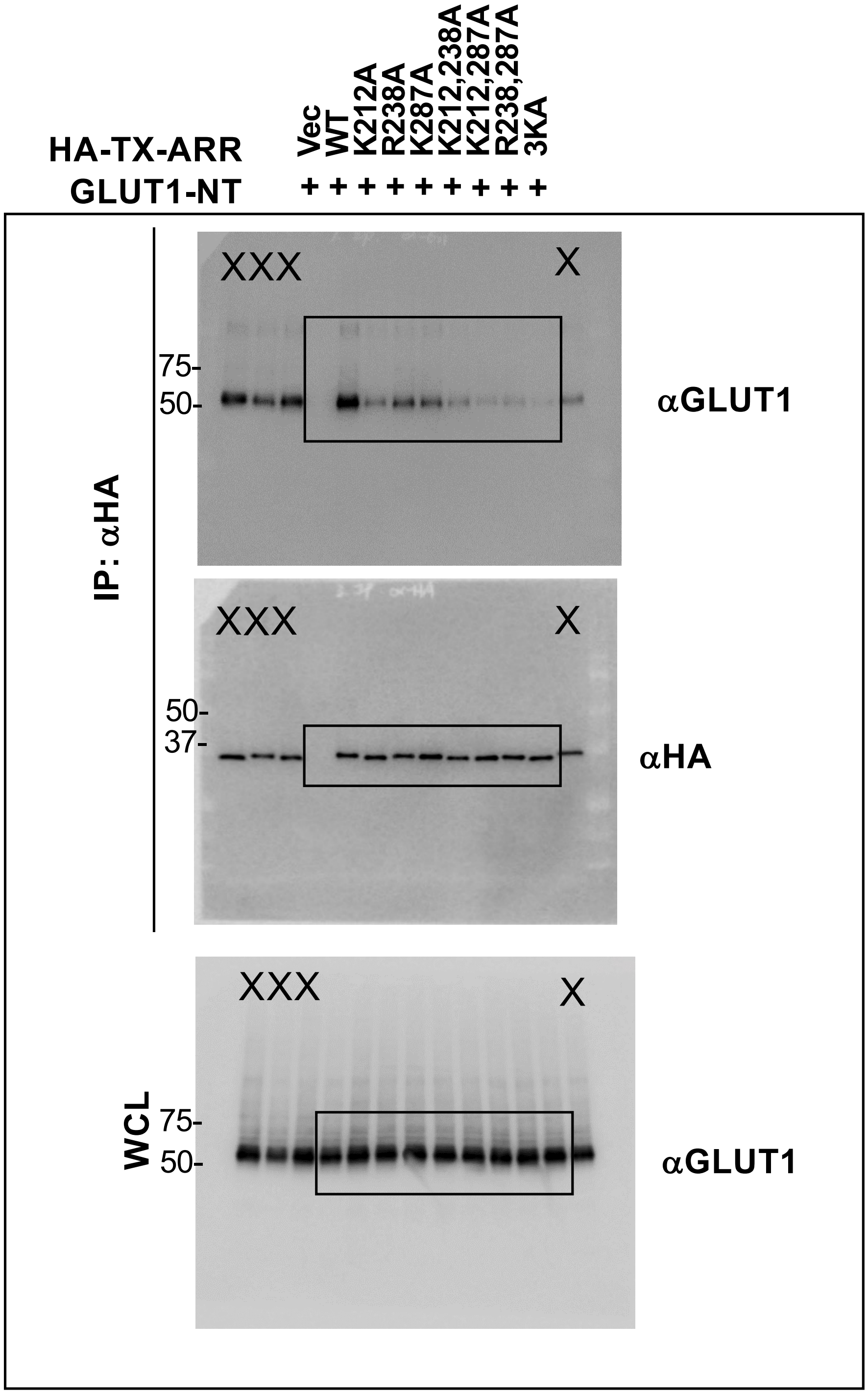
HA-TX-ARR
GLUT1-NT
Vec
WT
K212A
R238A
K287A
K212,238A
K212,287A
R238,287A
3KA
+ + + + + + + + +
IP: αHA
XXX
X
75-
50-
αGLUT1
XXX
X
50-
37-
αHA
WCL
XXX
X
75-
50-
αGLUT1

7C

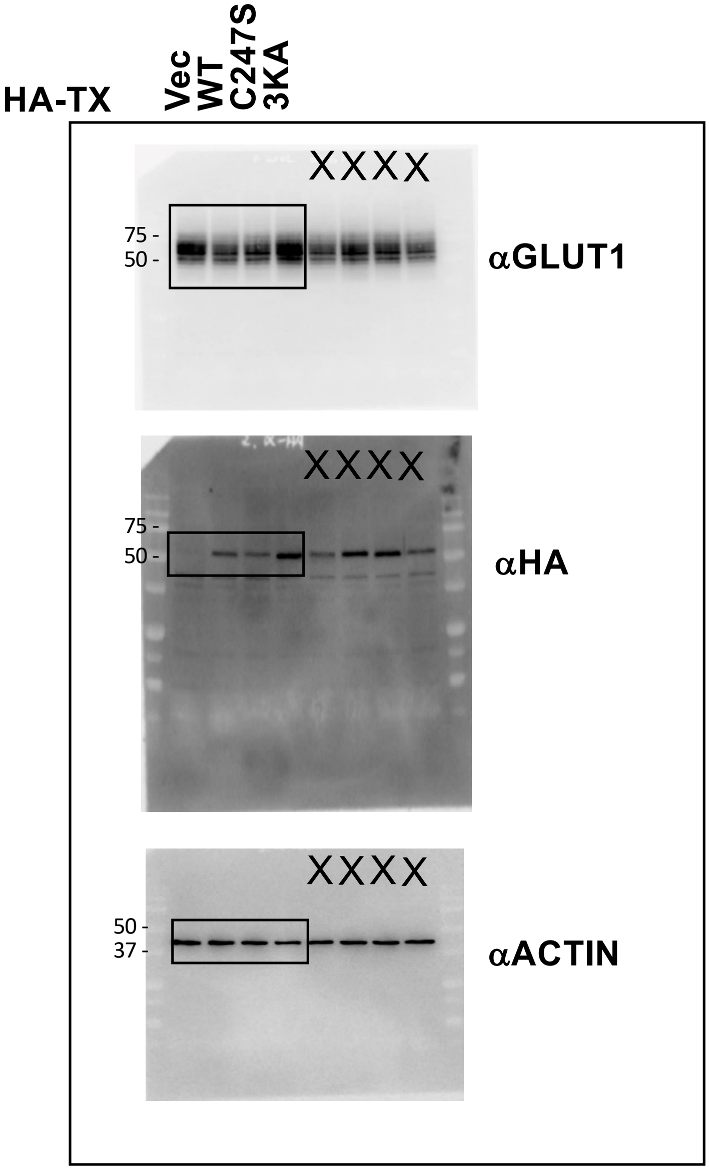

HA-TX
Vec
WT
C247S
3KA
XXXX
75 -
50 -
αGLUT1
XXXX
75 -
50 -
αHA
XXXX
50 -
37 -
αACTIN

S1D

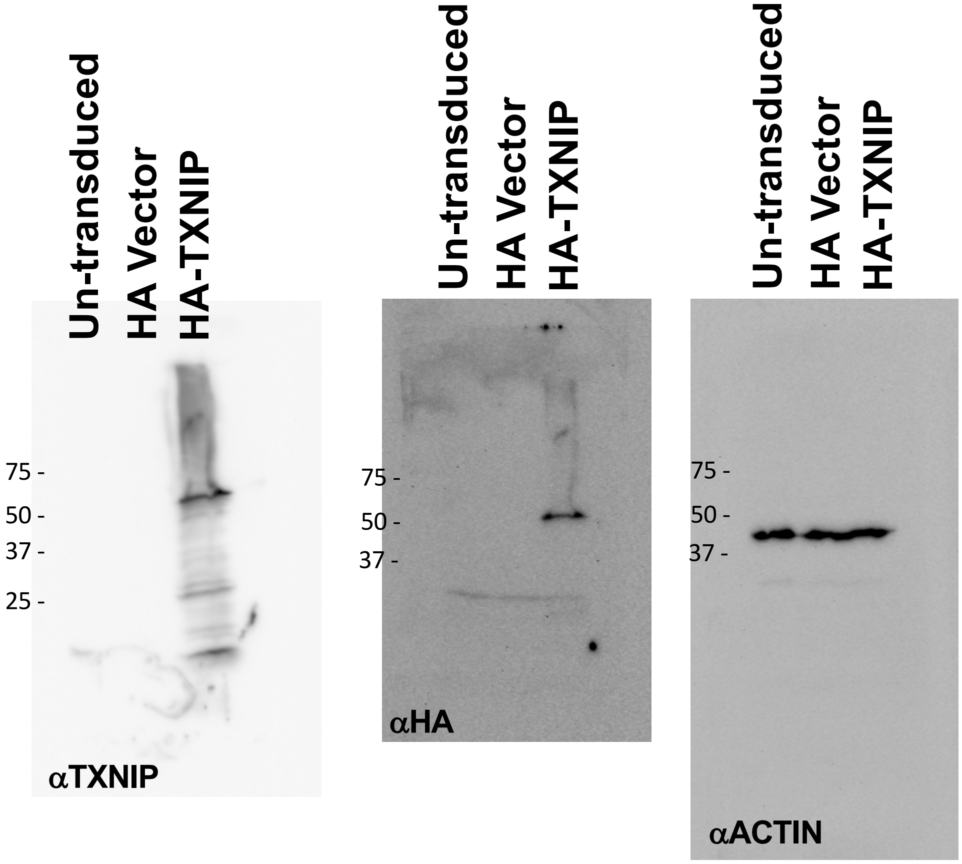
Un-transduced
HA Vector
HA-TXNIP
75 -
50 -
37 -
25 -
αTXNIP
Un-transduced
HA Vector
HA-TXNIP
75 -
50 -
37 -
αHA
Un-transduced
HA Vector
HA-TXNIP
75 -
50 -
37 -
αACTIN

S2B

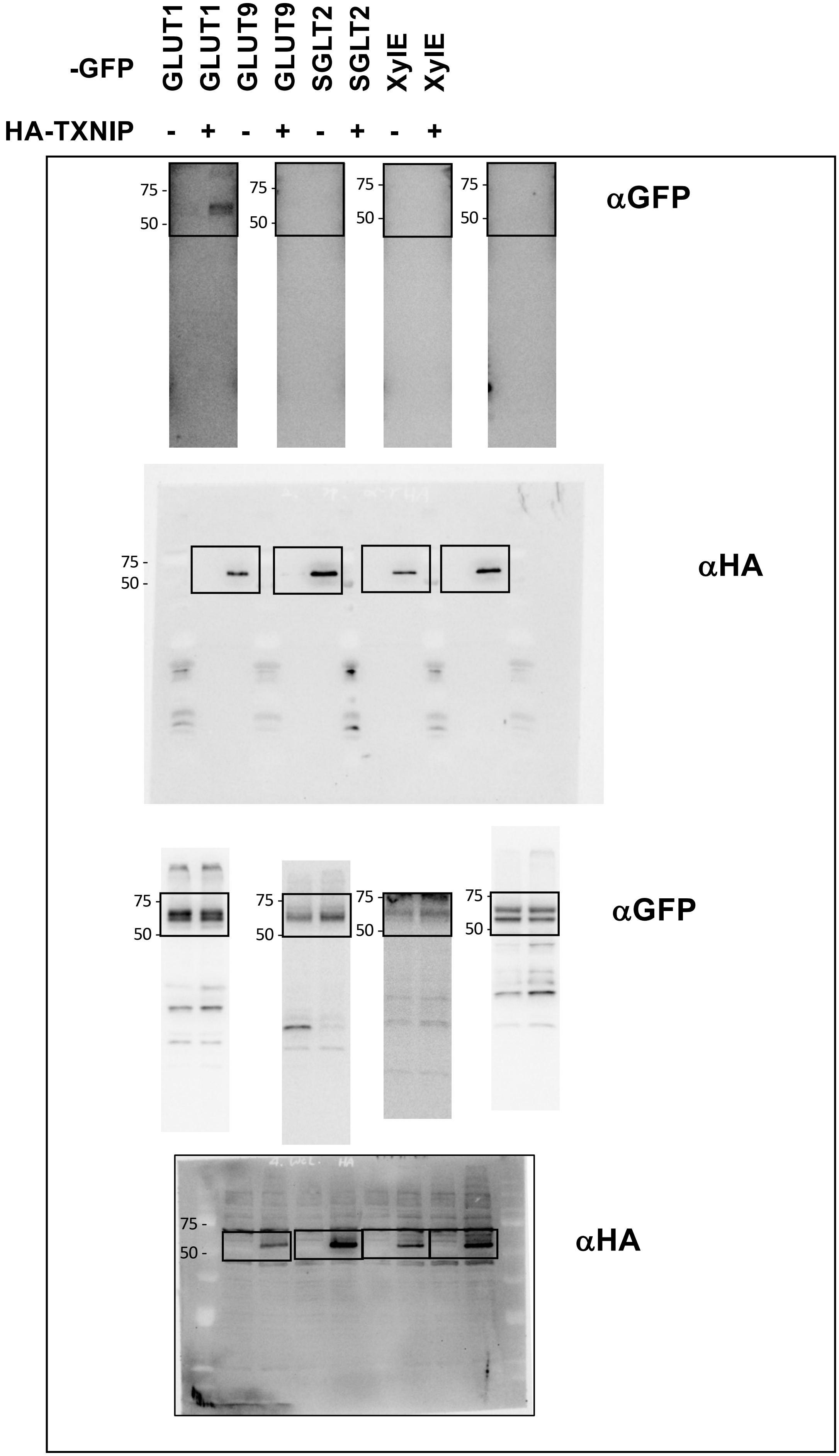
-GFP
GLUT1 GLUT1 GLUT9 GLUT9 SGLT2 SGLT2 XylE XylE
HA-TXNIP - + - + - + - +
75
50
αGFP
αHA
αGFP
αHA

S4B

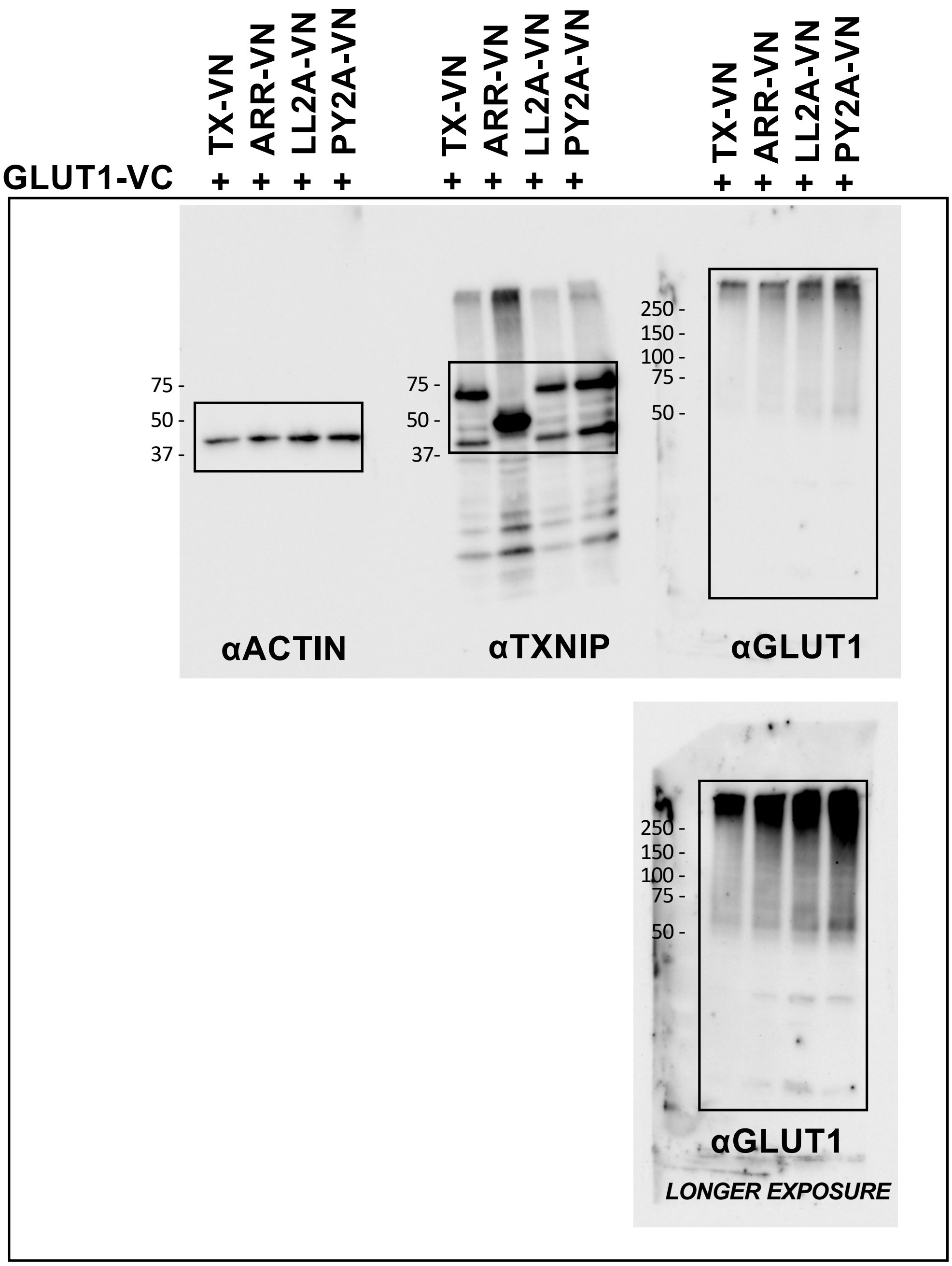
GLUT1-VC
TX-VN
ARR-VN
LL2A-VN
PY2A-VN
75 -
50 -
37 -
250 -
150 -
100 -
αACTIN
αTXNIP
αGLUT1
αGLUT1
LONGER EXPOSURE

S5A

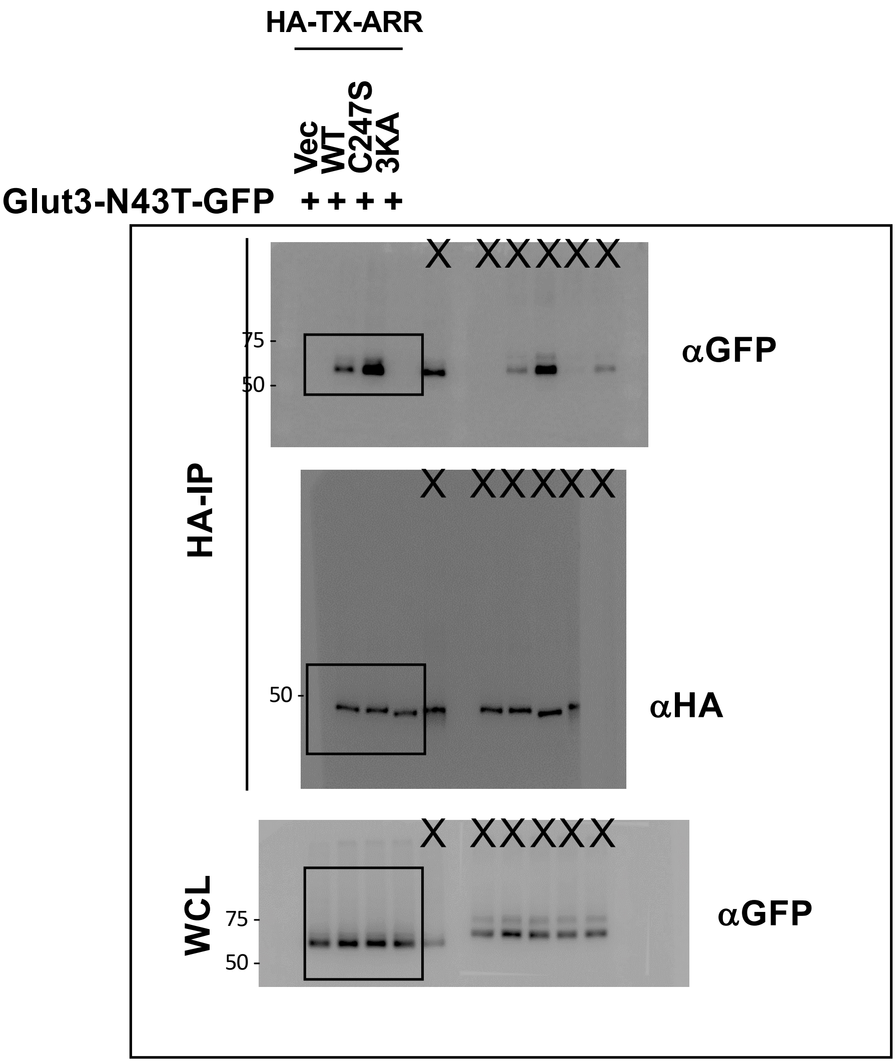
HA-TX-ARR
Vec
WT
C247S
3KA
Glut3-N43T-GFP + + + +
HA-IP
WCL
αGFP
αHA
αGFP
75
50
50
75
50

S5B

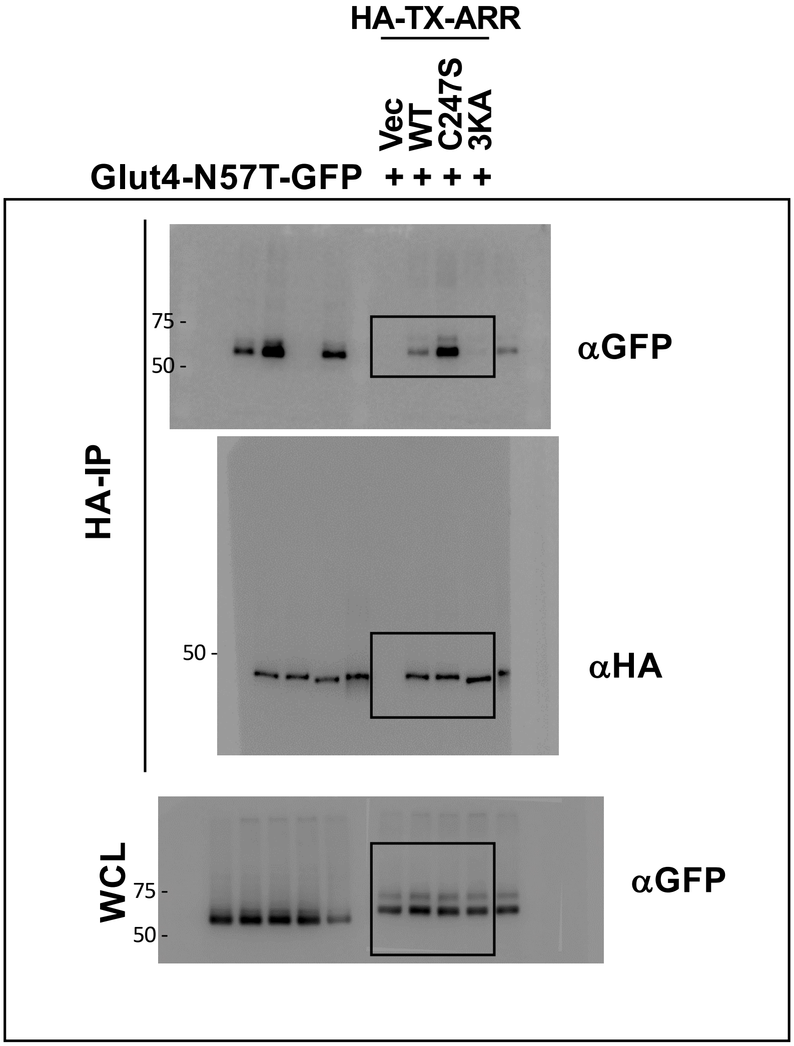
HA-TX-ARR
Vec
WT
C247S
3KA
Glut4-N57T-GFP + + + +
HA-IP
75 -
50 -
αGFP
50 -
αHA
WCL
75 -
50 -
αGFP
